# Predicting host species susceptibility to influenza viruses and coronaviruses using genome data and machine learning: a scoping review

**DOI:** 10.3389/fvets.2024.1358028

**Published:** 2024-09-25

**Authors:** Famke Alberts, Olaf Berke, Leilani Rocha, Sheila Keay, Grazieli Maboni, Zvonimir Poljak

**Affiliations:** ^1^Department of Population Medicine, Ontario Veterinary College, University of Guelph, Guelph, ON, Canada; ^2^Centre for Public Health and Zoonoses, University of Guelph, Guelph, ON, Canada; ^3^Centre for Advancing Responsible and Ethical Artificial Intelligence, University of Guelph, Guelph, ON, Canada; ^4^Athens Veterinary Diagnostic Laboratory, Department of Infectious Diseases, College of Veterinary Medicine, University of Georgia, Athens, GA, United States

**Keywords:** influenza A viruses, coronaviruses, machine-learning, genome, scoping review, interspecies transmission, spillover

## Abstract

**Introduction:**

Predicting which species are susceptible to viruses (i.e., host range) is important for understanding and developing effective strategies to control viral outbreaks in both humans and animals. The use of machine learning and bioinformatic approaches to predict viral hosts has been expanded with advancements in *in-silico* techniques. We conducted a scoping review to identify the breadth of machine learning methods applied to influenza and coronavirus genome data for the identification of susceptible host species.

**Methods:**

The protocol for this scoping review is available at https://hdl.handle.net/10214/26112. Five online databases were searched, and 1,217 citations, published between January 2000 and May 2022, were obtained, and screened in duplicate for English language and *in-silico* research, covering the use of machine learning to identify susceptible species to viruses.

**Results:**

Fifty-three relevant publications were identified for data charting. The breadth of research was extensive including 32 different machine learning algorithms used in combination with 29 different feature selection methods and 43 different genome data input formats. There were 20 different methods used by authors to assess accuracy. Authors mostly used influenza viruses (*n* = 31/53 publications, 58.5%), however, more recent publications focused on coronaviruses and other viruses in combination with influenza viruses (*n* = 22/53, 41.5%). The susceptible animal groups authors most used were humans (*n* = 57/77 analyses, 74.0%), avian (*n* = 35/77 45.4%), and swine (*n* = 28/77, 36.4%). In total, 53 different hosts were used and, in most publications, data from multiple hosts was used.

**Discussion:**

The main gaps in research were a lack of standardized reporting of methodology and the use of broad host categories for classification. Overall, approaches to viral host identification using machine learning were diverse and extensive.

## 1 Introduction

**Figure 1:**
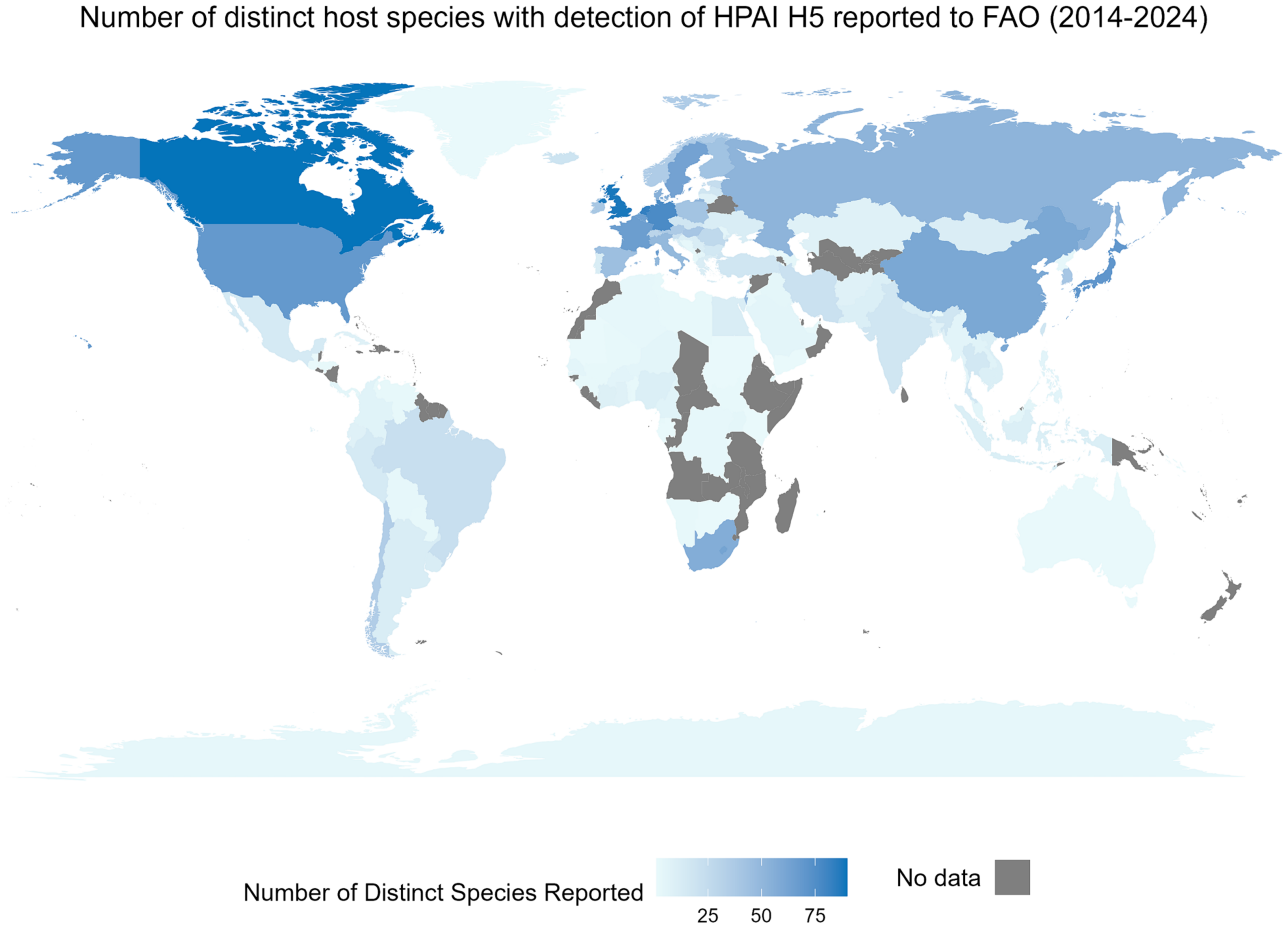
The global distribution of a country-level number of distinct non-human species with an event of highly pathogenic H5 influenza detection between January 2014 and May 2024. An event is defined as a nationally confirmed influenza finding in animals or humans. This includes outbreaks on farms, village or commune level, cases in wildlife or humans, or positive surveillance findings in animals (3). This figure includes only non-human events. Data were obtained from the Food and Agricultural Organization (FAO) EMPRES-i dataset on May 29, 2024. Data includes all available HPAI H5 information from FAO. Species were reported as reported by the FAO and were not altered, whereas territory and country names were modified in the FAO’s dataset when needed to allow for complete joining between the map and the attribute data.

Interspecies transmission of viruses is an ever-present risk to the well-being and health of humans and animals and to the vitality of agricultural practices (1, 2). Recent examples of the impact of these cross-species events within the One Health concept include the SARS-CoV-2 and H1N1 pandemics and the emergence of West Nile virus in North America, Severe Acute Respiratory Syndrome (SARS), and Middle East Respiratory Syndrome (MERS). An illustration of the global impact of these cross-species events is shown through the number of unique non-human species with reported infections of highly pathogenic H5 avian influenza at the country level available through the Food and Agriculture Organization (FAO) (Figure 1). To understand and mitigate respective events, knowledge of a pathogen’s susceptible species (i.e., host range) is essential (4). Susceptible species refers to the set of host species a distinct pathogen can infect; susceptibility is dependent on several factors including, but not limited to the nature of the virus, opportunity for spillover events, involvement of vectors and vector transmission pathways, etc. (5). Determination of susceptible species is challenging due to the mutable nature of viruses and the influences of both abiotic and biotic interactions (6, 7). Traditionally, the susceptibility of species is assessed using empirical evidence derived from methods such as laboratory testing, surveillance, and other epidemiological methods including phylogenetic analysis (8). Methods applying machine learning for prediction of the susceptible species may be beneficial for outbreak preparedness and response planning for outbreaks (7, 9). *In silico* approaches can enhance the ability to obtain results much faster and at a lower cost, while still being capable of analyzing complex data patterns and enhancing predictive accuracy (10, 11). Here we conduct a scoping review to characterize the current body of evidence surrounding these techniques. Influenza viruses and coronaviruses were the focus of this review because of their public health relevance and because of their known propensity for a broad range of susceptible species.

Two prior syntheses on this topic have been published. Abd-Alrazaq et al. (12) conducted a scoping review on the uses of artificial intelligence (AI) during the SARS-CoV-2 pandemic and identified three publications involving the use of AI to predict potential hosts of SARS-CoV-2. In a systematic review of machine learning used for predicting influenza phenotype, Borkenhagen et al. (13) identified eleven publications where machine learning was used to identify determinants of host tropism (infection specificity) of influenza virus and seven where machine learning was used to perform host prediction. Here we updated their searches to capture recent work, expanded on the scope of these reviews to include both coronaviruses and influenza viruses, and focused the review topic on the use of machine learning for predicting susceptible host species.

The goal of this scoping review was to address the research question: “What machine learning methods have been applied to influenza virus and coronavirus genome data for identification of potential reservoirs?” The body of evidence was characterized by types of viruses, genome types, types of analyses and machine learning classifiers applied.

## 2 Methods

### 2.1 Protocol and registration

The protocol for this review titled “The Use of Machine Learning and Predictive Modelling Methods in the Identification of Hosts for Viral Infections: Scoping Review Protocol” was prepared using the Preferred Reporting Items for Systematic Reviews and Meta-Analyses Extension for Scoping Reviews (PRISMA-ScR) reporting guidelines (14). The protocol was posted *a priori* (i.e., before study commencement) on the University of Guelph Institutional Repository (The Atrium, https://hdl.handle.net/10214/26112) on July 28th, 2021 (Supplementary material S2).

After the publication of the protocol, the following modifications were made:

1. Inclusion criteria for the publication year was extended to 2022, because an updated search was additionally performed on May 4th, 2022.
2. The study deduplication process, using Mendeley reference management software, was additionally performed using Distiller-SR (^©^2023 Systematic Review Software by Evidence Partners) (15).
3. Title and abstract screening at the first level was split into a level 1A and 1B because publications missing abstract metadata upon initial upload needed to be managed differently. To account for missing metadata, the first question in level 1A identified if the abstract was included in the metadata. If the abstract was not available upon the initial upload, the publication was moved to 1B where the abstract was then manually uploaded before further screening. All other screening questions remained the same.
4. Upon completion of pre-testing two publications, data charting was adjusted as follows:
  - Data charting was performed at the analysis-level rather than the publication-level (i.e., information from all analyses within a publication was charted into the number of analyses in a publication) to account for situations where publications contained more than one analysis.
  - Journal information and author-stated limitations were not charted.
  - For “data sources,” only the name of the data source for genome databases was collected.
  - In “data outcomes,” only the accuracy method and information regarding the use of predictive probabilities was collected.

### 2.2 Eligibility criteria

To be eligible for inclusion, publications had to be full-text English language publications of primary research from any geographic location and published between January 2000 to May 4th, 2022. The approaches outlined in the objectives of this review were not relevant prior to the introduction of whole genome sequencing and the advancement of machine learning techniques which, at the earliest, would have started being published in 2000. Eligible publications had to be primary research investigating the susceptible species of coronaviruses or influenza viruses singularly or in combination with other viruses. Conference proceedings were considered because computer science and engineering conference proceedings often meet indexing requirements as stand-alone publications and are fully searchable in major bibliographic databases. All genera of coronavirus and influenza virus were considered as each has been documented as capable of transmission in multiple host species; both are global public health and agricultural priority pathogens and have been extensively surveyed genomically (16). Eligible research included the use of machine learning techniques on any genomic data for the purpose of understanding or predicting influenza virus or coronavirus host-range or transmission.

### 2.3 Information sources

On July 29th, 2021, and May 4th, 2022, six bibliographic databases were searched through five bibliometric platforms (Table 1). Proceedings from three conferences were hand-searched (Table 2), as were references of all publications identified as relevant.

**Table 1:** Bibliographic databases and platforms (vendor interfaces) that were searched.

Platform (vendor interface)	Database
Clarivate	Web of Science Core Collection
Elsevier	Engineering Village-Inspec and Compendex
National Center for Biotechnology Information (NCBI)	PubMed—including MEDLINE (National Library of Medicine biomedical database of citations and abstracts indexed using MeSH thesaurus)
Ovid Technologies Inc.	MEDLINE
ProQuest	Coronavirus Research Database

**Table 2:** Hand-searched conference proceedings.

Conference	Proceeding availability
Computational Intelligence Methods for Bioinformatics and Biostatistics (CIBB)	Annual international conference. Selected conference publications are available for 2008–2019
International Conference on Computational Biology and Bioinformatics (ICCBB)	Annual international conference. Titles and full-text conference publications are available for 2017–2020
Intelligent Systems for Molecular Biology (ISMB)	Annual international conference. Titles and full proceedings are available for 1993–2020

The search string for Web of Science was formatted as follows:((TS = (zoono* or between-species transmission or host range or cross-species transmission or pathogen spillover or spillover or host tropism or host specificity or reservoir)
AND
TS = (machine learning or big data or convolution neural network or deep learning or network analysis or bioinformatics or predictive model* or unsupervised learning or supervised learning or semi-supervised learning or active learning or algorithm* or ai or artificial intelligence))
AND
TS = (influenza* or Orthomyxovir* or flu or Coronavir* or covid or IAV)
)


See also Supplementary Table S1 for the search strings for all the bibliographic databases.

### 2.4 Selection of sources of evidence (relevance screening), and data charting process

Citation metadata from the search output was compiled and deduplicated using Mendeley reference management software (15). Citation metadata were then uploaded to Distiller-SR (^©^2023) software package and deduplicated once again. Relevance screening was performed in three stages (levels 1A, 1B, and 2) using forms built in Distiller-SR (^©^2023). Levels 1A and 1B screening forms were identical but, level 1B was only for references found in level 1A screening to be missing abstract metadata and therefore required manual entry of abstracts prior to level 1B. Levels 1A and 1B relevance screening was at the level of the title and abstract. Level 2 screening was done using full texts. Screening and data charting were performed by two reviewers working independently followed by conflict resolution. If an agreement could not be reached, a third reviewer was consulted. The authors of references were not contacted for additional information. Full texts were obtained using EndNote citation management software (17) and through hand searching and then uploaded to Distiller-SR (^©^2023). The forms used are outlined in Supplementary Table S2.

Data on the following characteristics were charted:

1. First author employment/research affiliation(s).
2. First author region/country affiliation(s).
3. Type of viral genome(s) used and sources of information.
4. Host(s) taxonomy.
5. Sequence processing technique(s).
6. Machine learning classifier information including algorithm, feature selection, and type of validation used.
7. Measure(s) of accuracy used for classifiers.
8. Software used.

### 2.5 Synthesis of results

Data were downloaded from Distiller-SR (^©^2023) into R using RStudio (18, 19) for cleaning, analysis, and creation of relevant figures and tables. In R, a network analysis was performed to analyze the relationship between feature selection methods and machine learning methods used. “Tidyverse” (20) and “igraph” (21) packages in R were used to create a network analysis from the co-occurrence matrix between feature selection methods and machine learning methods.

Data charting results were split into three main levels as a logical grouping of the important information from all publications. These levels included:

1. Publication metadata including author information.
2. Analysis level information (a publication could consist of multiple analyses).
3. Information about machine learning classifiers (an analysis could consist of multiple classifiers).

## 3 Results

**Figure 2:**
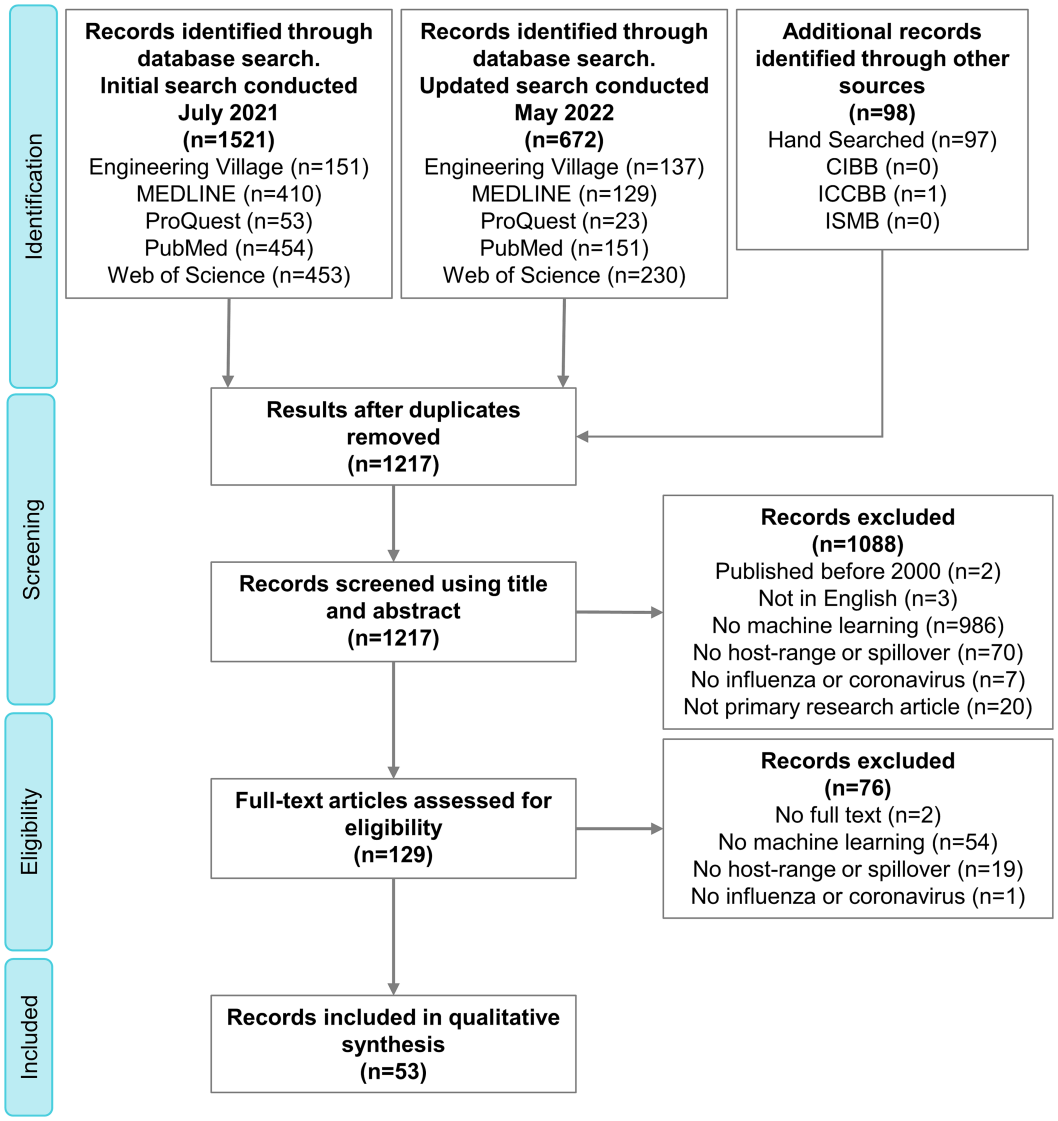
PRISMA flow diagram outlining the selection of relevant publications. PRISMA, Preferred Reporting Items for Systematic Reviews and Meta-Analyses; CIBB, Computational Intelligence Methods for Bioinformatics and Biostatistics; ICCBB, International Conference on Computational Biology and Bioinformatics; ISMB, Intelligent Systems for Molecular Biology.

The search strategy resulted in 1,217 citations, 53 of which were included in the final qualitative synthesis (Supplementary Table S3 and Supplementary material S1). The complete process of selection is shown in the PRISMA flow diagram (Figure 2).

### 3.1 Publication information

**Figure 3:**
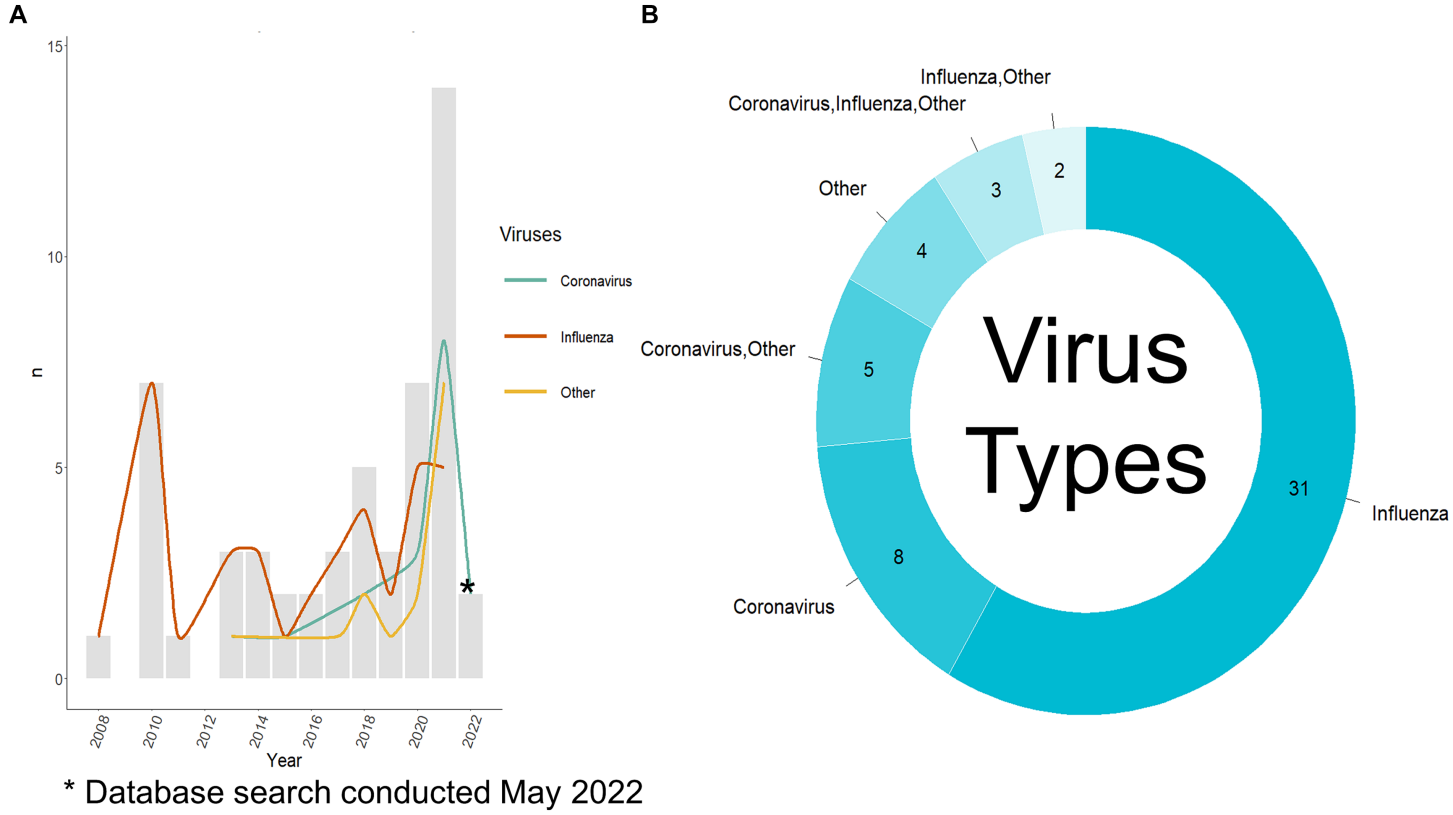
Viruses used per publication. **(A)** Viruses used per year per publication and the number of publications published per year. The search was conducted in May 2022 so there is not a complete record of publications from 2022. **(B)** Viruses used per publication, note: the publications where only others are listed did include coronavirus and influenza virus as part of a large group of viruses. Other viruses used are listed in Supplementary Table S5.

Fifty-three relevant publications were identified, all published between 2008 and 2022 (Figure 3A), of which, 41 were journal articles, nine were conference proceedings, and three were categorized as “other” due to their preprint status at the time of data charting. The first author’s country of affiliation was most commonly the United States of America (*n* = 16/53 publications, 30.2%), China (*n* = 15/53, 28.3%), and the United Kingdom (*n* = 8/53, 15.1%) (Table 3). First author institutional affiliation was most frequently a university (*n* = 45/53, 84.9%), national or sub-national government organizations (*n* = 10/53, 18.9%), non-governmental organizations (*n* = 1/53, 1.9%), and private research institutions (*n* = 1/53, 1.9%). The department(s) of the first author were grouped into general categories (Supplementary Table S4). The most common was computer science or a related field (*n* = 25/53, 47.2%), biological sciences (*n* = 12/53, 22.6%), health (*n* = 8/53, 15.1%), data science (*n* = 7/53, 13.2%), and microbiology (*n* = 5/53, 9.4%) (Table 3).

**Table 3:** General characteristics of publication information used in the selected publications.

Publication characteristic	Number of publications (*n* = 53)^a^	Percentage (%)
Publication type		
Conference proceeding	9	17.0
Journal article	41	77.4
Other	3	5.6
First author country		
United States of America	16	30.2
China	15	28.3
United Kingdom	8	15.1
Egypt	4	7.5
Singapore	4	7.5
Germany	2	3.8
Canada	1	1.9
Cyprus	1	1.9
Iran	1	1.9
Israel	1	1.9
South Korea	1	1.9
First author institutional affiliation		
University	45	84.9
(Sub-)national government organization	10	18.9
Non-governmental organization	1	1.9
Private research institute	1	1.9
First author department^b^		
Computer Science	25	47.2
Biological Sciences	12	22.6
Health	8	15.1
Data Science	7	13.2
Microbiology	5	9.4
Mathematics and Statistics	3	5.7
Engineering	2	3.8
Agriculture	1	1.9
Epidemiology	1	1.9
Not stated	1	1.9

a Some publications may belong to multiple categories.

b Supplementary Table S4 includes departments included in each department category.

### 3.2 Analysis level information

An analysis was defined as; the same training and test source population was used with one or more classifiers in one or more input formats. A total of 77 analyses were reported across the 53 publications. To accurately chart all data, publications were split into the number of analyses they contained. The median number of analyses per publication was one. The range of analyses within the publications was one to five. Across publications, the general process for each analysis was host and virus selection, obtaining relevant genome data, processing these data into the appropriate form for input into the classifier, predicting the host or spillover of the viral genome using the classifier, and finally generating a measure of accuracy.

#### 3.2.1 Host and virus selection

The first step of the analysis process was selecting which hosts and virus types were to be used. Most authors used only influenza virus data (*n* = 31/53 publications, 58.5%, *n* = 40/77 analyses, 51.9%), 15.1% used only coronavirus data (*n* = 8/53, 15.1%, *n* = 18/77, 23.4%), and the remaining publications (*n* = 14/53, 26.4%, *n* = 19/77, 24.7%) used a combination of influenza viruses, coronaviruses, and other viruses (Figure 3B). The most common other virus included were of the *Rhabdoviridae* family (*n* = 10/17 analyses that used other viruses, 58.8%), specifically, rabies (*n* = 3/17, 17.6%), followed by multiple other viral species, families, and genera (as stated by the authors) (Supplementary Table S5). Bartoszewicz et al. (22) used almost all available viruses on the Virus-Host Database (7,503 species) and Sutanto and Turcotte (23) used almost all available RNA viruses on NCBI Virus. Figure 3A depicts the type of viruses used per publication as split into a yearly count when compared to the total number of publications published that year. Prior to 2012, all the publications focused on influenza viruses. Aguas and Ferguson (24) were the first to incorporate coronaviruses and other viruses (*Flavivirus* and *Alphavirus* genera, *Calciviridae* and *Paramyxoviridae* families) in their analysis. Recent publications show a shift to greater incorporation of coronaviruses among other viruses with a focus on investigating a variety of viruses in one publication (9, 22–33).

Analyses considered in this scoping review were conducted using various subsets of available genome data and implied or stated inclusion criteria. This was addressed through charting of the highest taxonomic or other classification level for the influenza viruses and coronaviruses. Forty-two analyses (*n* = 42/77, 55.8%) used influenza viruses. One analysis used the genome data of viruses from the family *Orthomyxoviridae*, whereas the majority of analyses (*n* = 38/42 influenza analyses, 90.5%) used only viruses from influenza A type, one analysis used only viruses of the H7N9 subtype, and two analyses did not clearly specify which taxonomic level was used. Twenty-three (*n* = 23/77 analyses, 41.5%) analyses used coronaviruses, 13 of these used genomes of viruses from the entire family *Coronaviridae*, one was the subfamily *Orthocoronavirinae*, five specified the level of species but did not specify which species, five were SARS-CoV-2, three were MERS-CoV, three were SARS-CoV-1, and two did not state the taxonomic level used. Eight of the coronavirus analyses used multiple distinct taxonomic levels within the same analysis and were therefore counted multiple times in the latter count of coronaviruses; 7 analyses used 2 levels, and 1 analysis used 3 levels. Nineteen analyses (*n* = 19/77, 24.7%) used other viruses that were not influenza viruses or coronaviruses, either alone or in different combinations with influenza viruses and/or coronaviruses.

Various host types were studied, some classified only as human versus non-human (*n* = 8/77, 10.3%) (22, 26, 30, 33–36), 3 analyses (3.9%) used pandemic human as a host (24, 37, 38), 3 analyses (3.9%) used zoonotic as a host (39–41) and others made classifications including multiple taxonomic levels (e.g., species and genus) (*n* = 14/77, 18.2%) (42). Based on analyses with reported hosts, on average, 5.0 hosts were studied per analysis (min = 2, med = 3, max = 24). Publications focusing specifically on coronavirus studied on average 5.52 hosts per analysis (min = 2, med = 5, max = 21), and publications using specifically influenza viruses used an average of 3.07 hosts per analysis (min = 2, med = 3, max = 9). Hosts were charted by taxonomic level. The most common taxonomic group was species (*n* = 67/77, 85.7%). Definitions for species level were non-strict since some authors defined species to a non-species level (i.e., non-human, avian, bovine, etc.). The results for species level are in Supplementary Table S6. The three most common species classifications were human (*n* = 57/77, 74.0%), avian (*n* = 35/77, 45%), and swine (*n* = 28/77 36.4%). The remaining taxonomic levels were reported in Supplementary Table S7.

#### 3.2.2 Collation of genome data

A variety of techniques were used to obtain the appropriate genome sequences. Most authors only used viral genome sequences of the viruses they were interested in (*n* = 68/77 analyses, 88.3%). However, there were nine analyses (six publications) that used the host information to some capacity in combination with the viral genome. Mollentze et al. (30) and Bergner et al. (26) used the host information by calculating a human similarity statistic which was then used to assess the predictive probability of the virus to infect humans. Wardeh et al. (43) used mammalian phylogenetic, ecological, and geospatial traits to enhance their viral host informational traits. These traits were used in combination with viral genomics for classifier predictions of potential hosts of coronaviruses. Yang et al. (31) combined the host information from GenBank to map their viruses into 10 different host categories.

**Figure 4:**
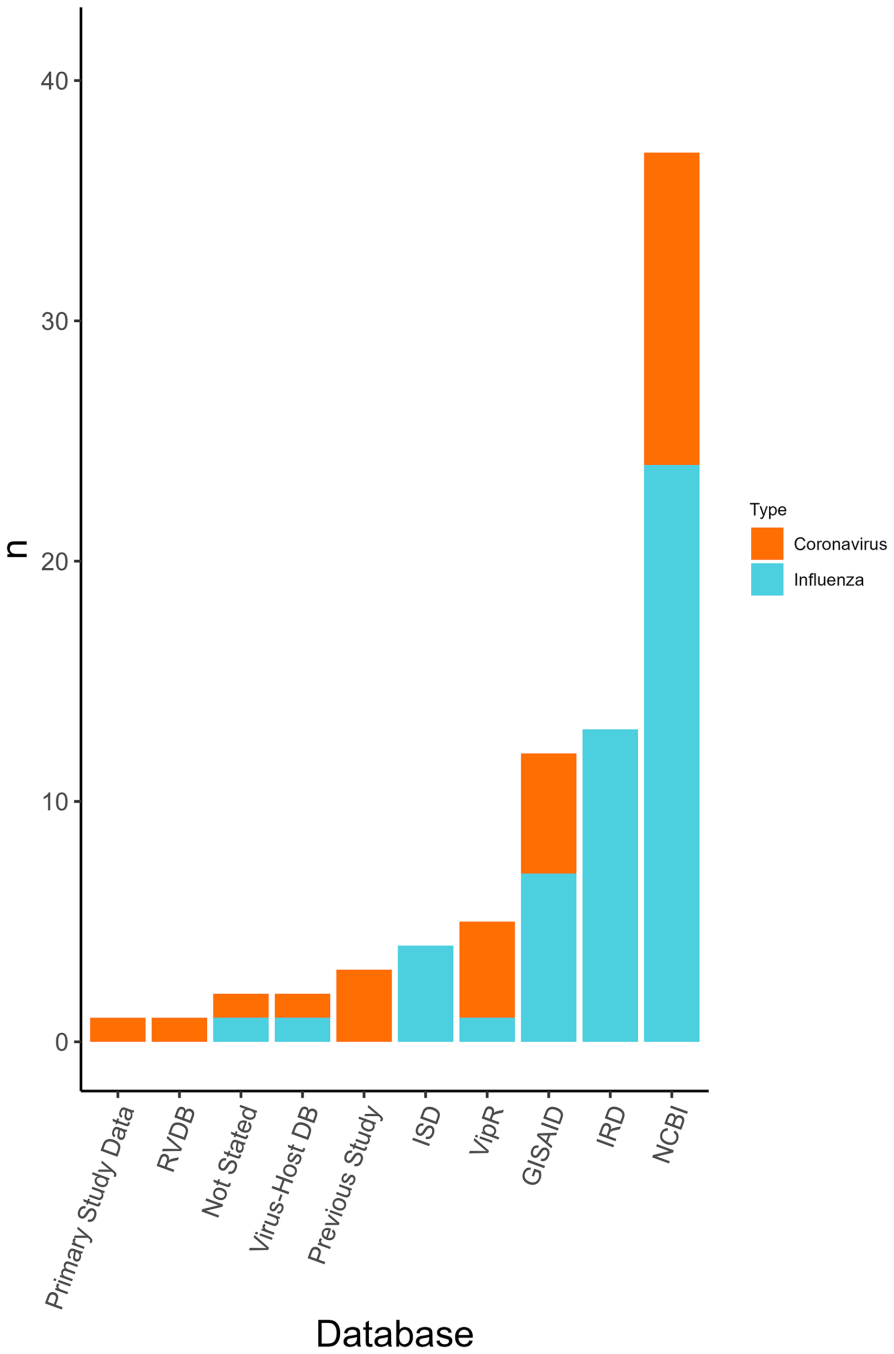
The databases used for obtaining viral genome sequences to be used within the classifiers and the number of analyses that used each database *(n)*. Displayed by the virus type the database was used for (i.e., influenza virus or coronavirus). Database type was not collected for “other” viruses only influenza viruses and coronaviruses. Some analyses may have used multiple databases. RVDB, Reference Viral Database; Virus-Host DB, Virus-Host Database; ISD, Influenza Sequence Database (merged with IRD); ViPR, Virus Pathogen Database and Analysis Resource [merged with IRD to become Bacterial and Viral Bioinformatics Resource Center (BV-BRC)]; GISAID, Global Initiative on Sharing Avian Influenza Data; IRD, Influenza Research Database [merged with ViPR to become Bacterial and Viral Bioinformatics Resource Center (BV-BRC)]; NCBI, National Center for Biotechnology Information.

Viral genomes were used in all the publications and the most common source for coronavirus and influenza virus sequence data was NCBI (*n* = 36/77 analyses, 46.8%), Influenza Research Database (IRD) (*n* = 13/77, 16.9%), and Global Initiative on Sharing Avian Influenza Data (GISAID) (*n* = 7/77, 9.1%) (Figure 4). Coronavirus genome data were used in the form of the whole genome (*n* = 6/23 coronavirus analyses, 26.0%), the spike gene (*n* = 8/23, 34.8%), and was not stated in the remainder (*n* = 9/23, 39.1%). Influenza genome data were charted using the eight RNA segments PB2, PB1, PA, NP, HA, NA, M, and NS. In some analyses the proteins used were specified from within the segments, however, this information was not charted. Segments were either used separately or concatenated. The whole genome (*n* = 18/42 influenza analyses, 42.8%), partial genomes (i.e., a variety of combinations of the eight segments) (*n* = 11/42, 26.2%), only HA (*n* = 8/42, 19.0%), only PB1 (*n* = 1/42, 2.4%), or PB2 (*n* = 1/42, 2.4%) were used. In three studies, it was not revealed whether a whole or partial genome was used.

#### 3.2.3 Pre-processing genome data

**Figure 5:**
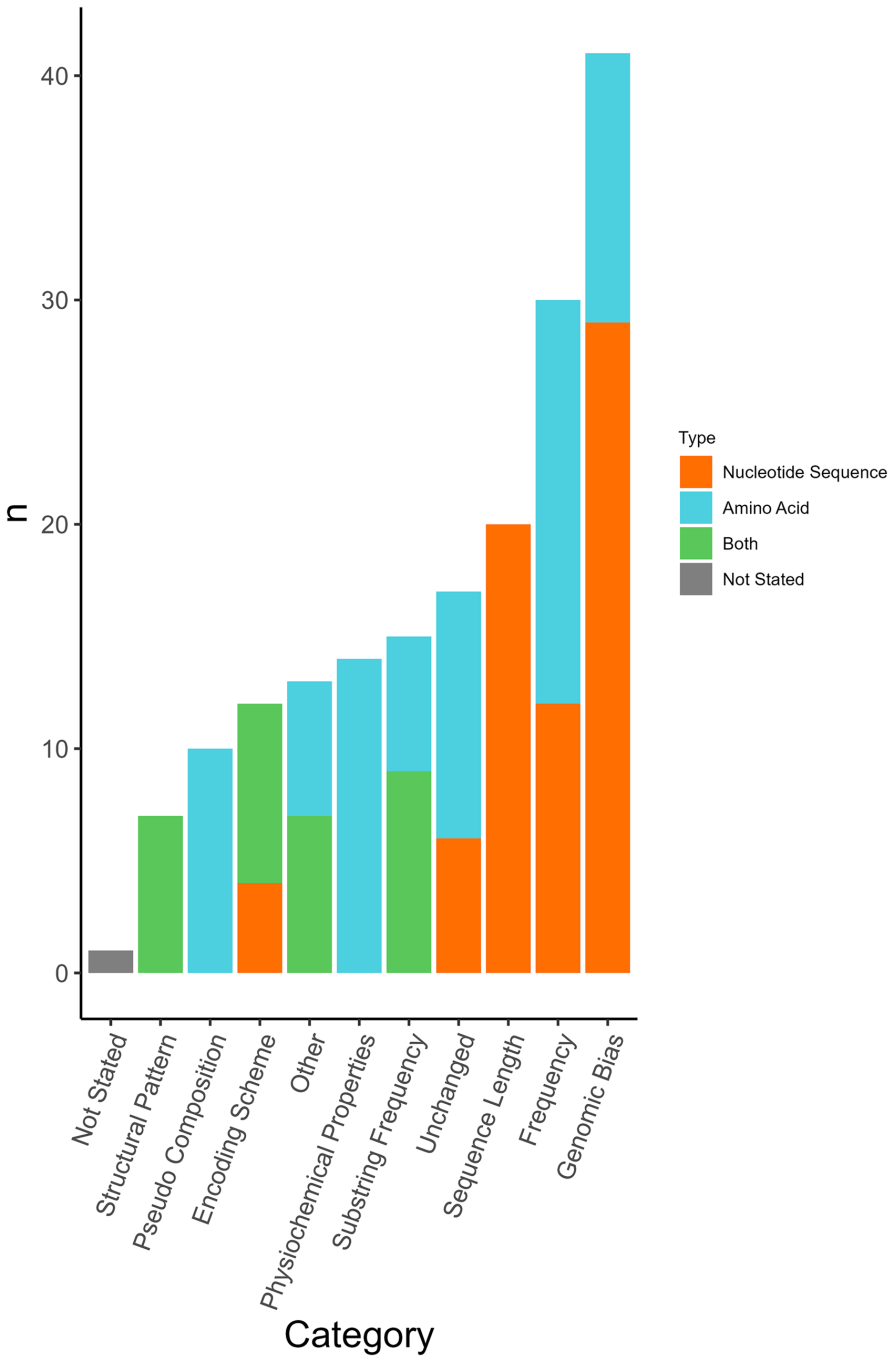
Sequence format transformations applied per analysis. Some analyses used multiple transformations. In some analyses, transformations were applied to both nucleotide sequence and amino acid sequence. Each category contains multiple different formats amalgamated into one category (Supplementary Table S8). The classification was determined by the most common sequence format used. The format used in the corresponding figure was determined based on whether nucleotide sequence, amino acid sequence, or both were selected in the corresponding input sequence question if no selection was made the classification defaulted to that used in Supplementary Table S8.

Viral genome sequences were preprocessed prior to use in machine learning algorithm(s). Transformations were applied to both the nucleotide sequences and amino acid sequences and then used as input features in the machine-learning classifier. Forty-one types of feature transformations were used as well as the untransformed nucleotide sequence or amino acid sequence (Supplementary Table S8). The 41 types of feature transformations were categorized into 10 categories (Figure 5). Authors commonly used a combination of multiple sequence formats/transformations to curate their feature library for each sequence (*n* = 57/77 analyses, 74.0%). Use of an unchanged sequence as an input (unchanged nucleotide = 6/77 analyses, 7.8%, unchanged amino acid = 11/77, 14.3%) was reported in 22% (*n* = 17/77) of analyses.

Genomic bias accounts for the proportionality of bases or codons in the form of the sequence length, dinucleotide frequencies, and mononucleotide frequency amongst others. Bias scores are calculated in several ways most often concerning expected or favoured compositional frequencies (44). Forty-one analyses (53.2%) incorporated one or more forms of genomic bias transformation. This transformation was applied to both nucleotide sequences (*n* = 29/77, 37.7%) and amino acid sequences (*n* = 12/77, 15.6%). Frequency (*n* = 30/77, 39.0%) and substring frequency (*n* = 15/77, 19.5%) were transformations to account for various frequencies and sequence compositions. Most frequency measures were implemented as a count, however, a study employed word embedding methods to factor in the relation between words rather than strictly a count (45). Twenty-six percent (*n* = 20/77) of analyses transformed the sequence length, including adjustments such as adjusting all sequences to have the same length as the shortest sequence (29), generating short sequences (100–400 bp or 400–800 bp) to reduce the computational complexity (27), only using the receptor binding site of HA (38), amongst others. Pseudo-compositions were used in 10 analyses. Pseudo-composition incorporates features from the original sequence and features from pseudo-components such as in the calculation of order. Yerukala Sathipati et al. (36) performed an analysis on four different feature descriptors including pseudo-amino acid composition (PseAAC) with eight different classifiers. Physiochemical properties consider features such as hydrophobicity, isoelectric point, and molar mass and were also used as a feature descriptor in Yerukala et al. (36). Fourteen analyses used physiochemical properties; physiochemical property transformations were only applied to amino acid sequences. Seven (9.1%) analyses used structural patterns and the assessment of secondary structures association with a virus’s ability to infect a host. Sequence features categorized as “other” included phylogenetic neighbourhood assessment, assessment of similarity to human genome transcripts, and the output of truncated singular value decomposition. Similarity to the human genome was used as previously described (30). In 74% (*n* = 57/77) of analyses, multiple sequence formats were used either as a comparative method or a combined feature matrix.

#### 3.2.4 Software and programming languages used

Nine different software or programming languages were applied to the data, and the different software or programming languages used were categorized at the analysis level. The most common were R (*n* = 28/77 analyses, 36.4%), Python (*n* = 23/77, 29.9%), MATLAB (*n* = 10/77, 13.0%), Weka (*n* = 10/77, 13%), and Java (*n* = 3/77, 3.9%) (Table 4). In 18.2% (*n* = 14/77) of analyses, the authors did not reference the software or programming language used. In 20.8% (*n* = 16/77) of analyses, more than one software or programming language was used. The most applied combinations were a combination of R and Python (9, 25, 42, 46) (*n* = 7/77, 9.1%) or a combination of Weka and an additional software or programming language (*n* = 7/77, 9.1%) (36, 37, 47–50).

**Table 4:** Software/programming language used.

Software	Number of analyses (*n* = 77)^a^	Percentage (%)
R	28	36.4
Python	23	29.9
MATLAB	10	13.0
Weka	10	13.0
Java	3	3.9
LIBSVM	3	3.9
RapidMiner	1	1.3
Spark ML	1	1.3
SVM-Light	1	1.3
Not stated	14	18.2

a Some analyses may belong to multiple categories.

### 3.3 Classifier level information

The general characteristics of classifiers used in the selected publications are presented in Table 5. Each analysis used one or more classifiers. Classifiers used a form of feature selection, a machine learning algorithm, and a form of validation. In cases where an ensemble of classifiers was used each classifier within the ensemble was charted separately. During charting, we indicated that these classifiers were part of an ensemble. In total, there were 174 classifiers used, the median number of classifiers used per analysis was one, and the range of classifiers used was one to eight. Most of the classifiers used in separate analyses were newly trained classifiers making up 87.4% of classifiers (*n* = 152/174 classifiers). For the remainder of the classifiers, they had been used previously (*n* = 20/174, 11.5%), or it was unclear if the classifier had been used previously (*n* = 2/174, 1.1%). In cases where a classifier had been used previously, sometimes it was an expansion on previous research, and other times it was to compare a novel classifier to an already developed model.

**Table 5:** General characteristics of classifiers used in the selected publications.

Classifier characteristic	Number of classifiers (*n* = 174)	Percentage (%)
Was a novel classifier		
Yes	152	87.4
No	20	11.5
Unclear	2	1.1
Used more than one host to train the classifier		
Yes	168	96.5
No	2	1.1
Unclear	4	2.3
Used more than one viral group to train the classifier		
Yes	104	59.8
No	24	13.8
Unclear	46	26.4
Class of classifier		
Supervised	172	98.8
Unsupervised	2	1.1
Semi-supervised	0	0
Top 8 machine learning algorithm categories^a^		
Neural network	39	22.4
Random forest	34	19.5
Support vector machine	28	16.1
Classification tree	15	8.6
k-nearest neighbour	15	8.6
Gradient boosting machine	12	6.9
Naïve bayes	12	6.9
Logistic regression	11	6.3
The number of sequences used		
<100	5	2.9
100–1,000	58	33.3
1,000–10,000	56	32.2
10,000–100,000	28	16.1
100,000–1,000,000	17	9.8
>1,000,000	4	2.3
Not stated	6	3.4

a Categorizations listed in Supplementary Table S10.

More than one host was used as discrete levels of the input in 96.5% (*n* = 168/174) of classifiers. With four classifiers it was unclear whether multiple hosts were used, and with two classifiers only one host was used. Bergner et al. (26) only used viruses derived from the saliva and feces of vampire bat colonies and assessed zoonotic potential by comparing these viruses with the known zoonotic potential of some viruses. The first analysis of Meroz et al. (38) predicted pandemic strains of H1N1, and the two comparative groups were human and pandemic human (human viruses with pandemic potential); in this case, only one species was used to train the classifier, but this one species was still split into multiple host groups. In 59.8% (*n* = 104/174) of the classifiers, multiple viral groups were used where viral groups were defined as the highest level of viral classification referred to within the publication. A viral group can be any taxonomic grouping. If an analysis specified only a singular group and did not compare multiple groups, it was recorded as not using multiple viral groups. In 26.4% (*n* = 46/174) of the classifiers, it was unclear if multiple viral groups were being used to train the classifiers and then in the remaining 13.8% (*n* = 24/174), only one viral group was used to train the classifiers.

The number of sequences used to train and test a classifier was charted by calculating the total amount of sequences used for one classifier (summation of all hosts), or in cases where the influenza segments were utilized, the maximum number of sequences per segment was charted. The average number of sequences used to train and test a classifier was 304,105, however, this number is skewed due to the large variability in the number of sequences used and extremes in the data (Table 5); the median number was 1902. The minimum number of sequences used was 17. Lee et al. (9), used 17 sequences of SARS-CoV2 and SARS-related viruses to predict reservoir hosts using a gradient boosting machine (GBM) classifier method in the second analysis that had been previously trained on a separate dataset within the first analysis reported in that publication. Bartoszewicz et al. (22) used the maximum number of sequences 14,242,329; however, these were not complete sequences of an entire virus, segment, or gene. Rather, reads of 150 base pairs were used and, such an approach was used for three classifiers.

Feature selection can improve the accuracy of a classifier and reduce computational requirements by removing redundancies and selecting more contributive features (51). In 52.9% (*n* = 92/174) of the classifiers, a feature selection was not used, or it was not stated. “Not used” and “not stated” were combined when referencing the feature selection method used since there was no explicit distinction for publications that explicitly chose to not use a feature selection. In all, 28 feature selection methods were used (Supplementary Table S9). The five most used were gradient-boosted classification trees as feature selection (*n* = 15/174, 8.6%), random forest as feature selection (*n* = 14/174, 8.0%), information gain (*n* = 11/174, 6.3%), single value decomposition (*n* = 8/174, 4.6%), ridge regression (*n* = 7/174, 4.0%) and in many the classifiers feature selection was not stated (*n* = 92/174, 52.9%). All feature selection types listed were used prior to input into the machine learning algorithm and were not a part of the classifier (i.e., when random forest was used as feature selection it was used in tandem with another machine learning algorithm).

**Figure 6:**
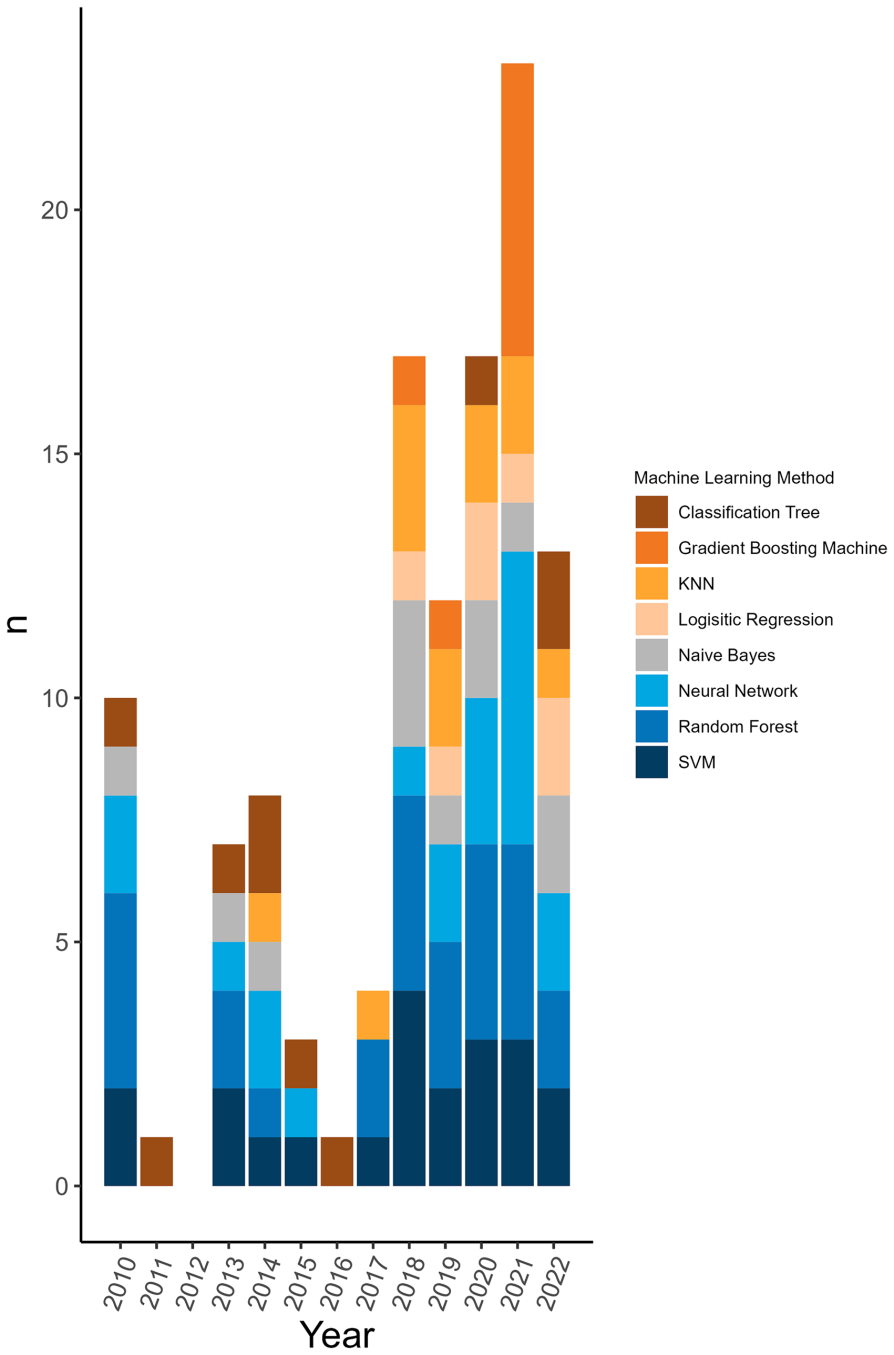
Top 8 machine learning algorithms used over time at the publication level. The representation of the top 8 machine learning algorithms used over time at the level of publication (i.e., if one publication used two different random forest classifiers it counts as random forest being used in a publication). Algorithms were displayed at the publication level rather than the classifier level to reflect the distribution of usage more accurately over time rather than representing a large change because one publication used one type of classifier multiple times.

**Figure 7:**
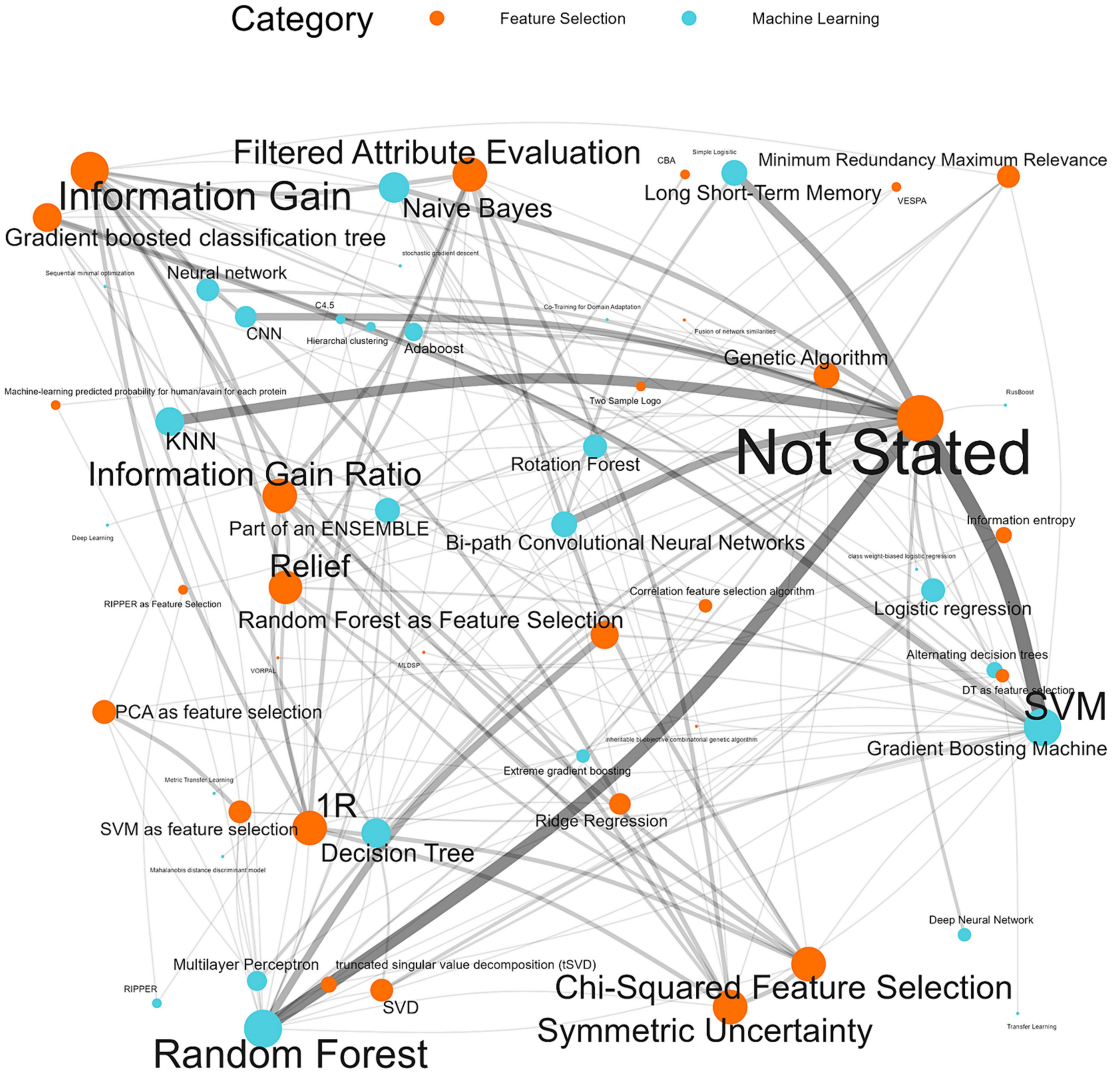
Machine learning algorithm and feature selection method network analysis. The thickness of the edges reflects the impact and frequency of that relationship within the network, similarly, the size of the node reflects the frequency of the method.

**Figure 8:**
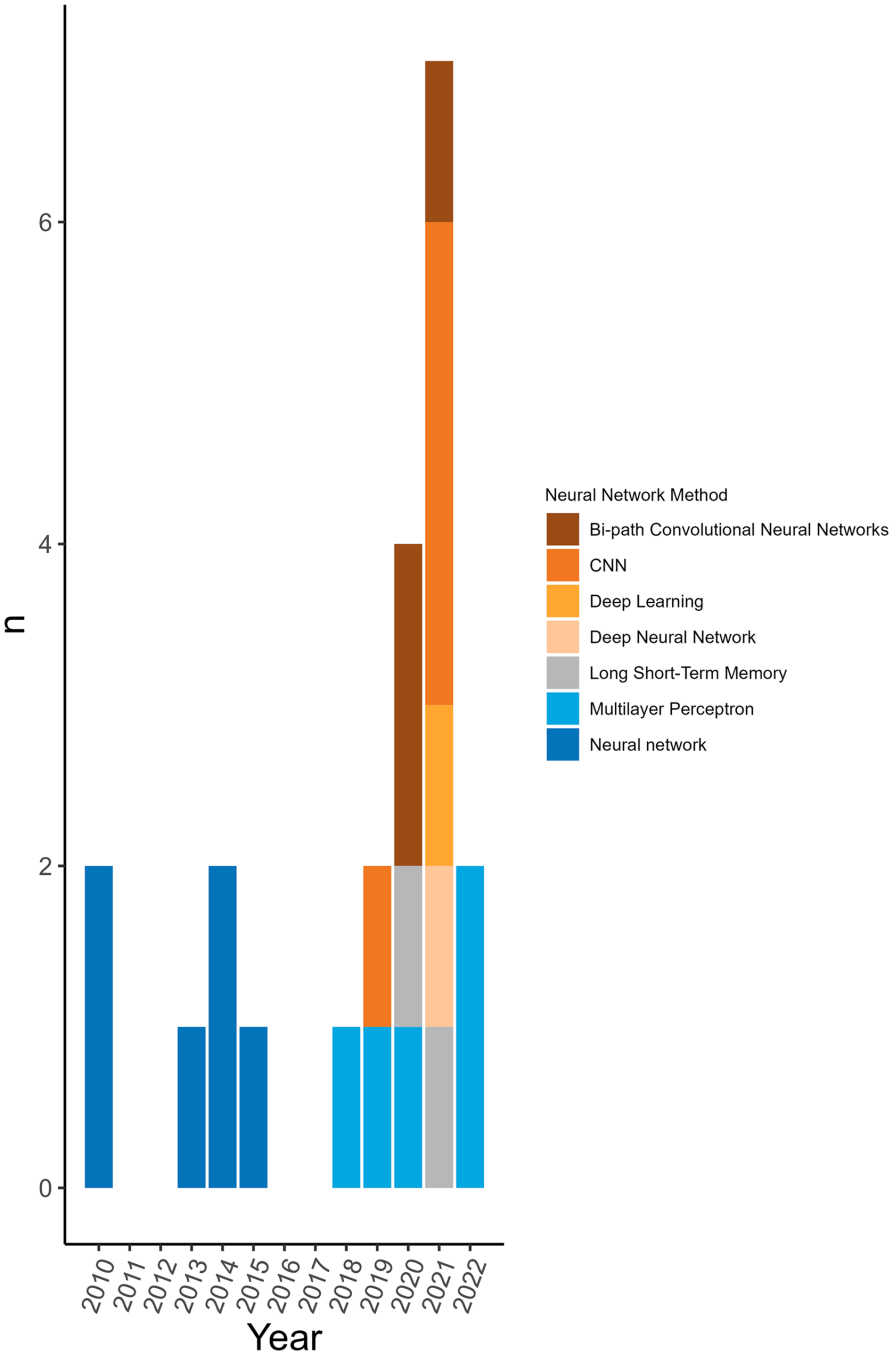
Neural network algorithms used over time. The neural networks used per publication over time to see the trend of distribution of type over time. Neural networks were not used every year.

98.8% (*n* = 172/174) of the machine learning algorithms used were supervised algorithms. Eng et al. (52) and Kou et al. (53) used an unsupervised hierarchical clustering approach. Kou’s et al. (53), approach was a descriptive analysis rather than the typical analytical analysis performed by other authors whose work was included in this review. The most used supervised learning algorithms were random forest (RF) (*n* = 31/174, 17.8%), support vector machine (SVM) (*n* = 28/174, 16.1%), k-nearest neighbour (KNN) (*n* = 15/174, 8.6%), classification tree (CT) (*n* = 12/174, 6.9%) and naïve Bayes (NB) (*n* = 12/174, 6.9%). In total, 31 different supervised algorithms were used (Supplementary Table S10). The machine learning categories presented in Figure 6; Table 5; Supplementary Table S10 encompass a variety of classifiers categorized into machine learning classes. A network analysis was performed to analyze the relationship between the feature selection method used and the machine learning algorithm used (Figure 7). From this network analysis, the most common occurrence was using RF, SVM, or KNN and not stating (or not using) a feature selection method (*n* = 37/174, 21.3%). Figure 8 displays the neural network classes over time.

Ensemble methods are machine learning approaches that use one or more models in combination (in an ensemble). Algorithms such as random forest and gradient boosting are examples of ensemble methods. Ensemble methods can also be used by combining the best results of multiple runs of a model (31, 43) or the best results of different input types such as k-mer length (34). Five classifiers from two publications were “part of an ensemble.” Kargarfard et al. (54) used CBA, RIPPER, and decision trees together in an ensemble for their final model. Kou et al. (46) used an ensemble of 64 random forest runs in their prediction algorithm.

Multiple types of validation were done for some classifiers (*n* = 96/174, 55.2%). The type of validation done was stated for 93.7% (*n* = 163/174) of classifiers. The most common types of validation were N-Fold Cross-Validation (*n* = 137/174, 78.7%), splitting the data into a training and validation dataset (*n* = 50/174, 28.7%) or training, optimization, and validation datasets (*n* = 34/174, 19.5%). There were nine classifiers (5.2%) where the authors did not split the datasets but rather used an entirely independent dataset. Other techniques that were used included simulation (*n* = 10/174, 5.8%), experimental validation (*n* = 9/174, 5.2%), and comparison to results from a previous classifier (*n* = 5/174, 2.9%) (Supplementary Table S11). Yang et al. (31) used the University of California, Irvine Medical Center medical benchmarks to validate their hybrid transfer classifier as a simulation of data before testing it on their coronavirus dataset. Another study using simulations was Bartoszewicz et al. (22) who simulated 250 bp Illumina reads to use as part of their validation technique.

The accuracy of the classifiers was assessed in 20 different ways and categorized on the analysis-level, not the classifier-level. The most common measures of accuracy reported included accuracy (*n* = 55/77 analyses, 71.4%), sensitivity (*n* = 31/77, 40.2%), specificity (*n* = 28/77, 36.3%), receiver operating characteristic (ROC) (*n* = 20/77, 26.0%), and the area under the curve (AUC) (*n* = 18/77, 23.4%). There were 15 other measures of accuracy as well (Supplementary Table S12) and some authors did not report the accuracy (*n* = 3/77, 3.9%). Accuracy was also used in terms of predictive probability in some cases. It was inferred that 21 analyses (27.3%) were using predictive probability to determine the most likely host, for three of the analyses it was unclear, and the remainder did not appear to be using predictive probability to determine the most likely host or for other types of inferences.

Twenty-five classifiers were identified in this scoping review as the best classifier used within a given analysis. This was determined based on what the authors deemed to be the best classifier or, if not stated, the classifier with the highest measure of accuracy. Analyses that only included one classifier were not assessed for this metric (*n* = 41/77 analyses, 53.2% or *n* = 41/174 classifiers, 23.0%). Of the 25 classifiers that were identified as the best, the most common feature selection was not used/not stated (*n* = 14/25, 56.0%), and the remaining 11 classifiers all used different feature selection methods. In analyses that identified the most accurate classifier, the most common machine learning algorithm class (category) identified was a neural network (bi-path, multiplayer perceptron, convolution) (*n* = 8/25, 32.0%), support vector machine (*n* = 6/25, 24.0%), k-nearest neighbour (*n* = 3/25, 12.0%), random forest (*n* = 3/25, 12.0%) and the remaining 5 classifiers used different machine learning algorithms.

## 4 Discussion

### 4.1 Summary of evidence

The main objectives of this review were to describe and categorize machine learning methods as a method for host prediction using viral sequences and genome data. There was a wide variety of approaches taken, and even where commonalities were found there were still many differences. This reflects the exploratory nature of computational data and the variety of approaches where the greatest limiting resources are time and data availability. Approaches differed not only comparatively to one another but also within publications. Many authors explored their data in multiple ways to configure the most effective predictive pipeline, and this was reflected by the number of analyses and the number of classifiers used per publication.

The main findings arose from the pre-processing of the sequences and the feature selection methods used prior to the machine learning algorithm. This is where the greatest disparity in methodological approaches was observed with 41 different pre-processing transformations applied to the genomic data. This shows the vast number of ways that data can be manipulated for the extraction of desired information. Publications showing a high degree of complexity incorporated measures related to phylogeny and host interaction. Commonly, each publication used more than one sequence pre-processing transformation (*n* = 57/77 analyses, 74.0%). Due to the large size of the feature libraries, feature selection tools were employed to reduce the dimensionality of the input database. Over half of the publications did not state or did not use a feature selection method. It may be that some classifiers did not require or benefit from a feature selection processing step to maximize classifier performance. Nonetheless, the rationale for using specific sequences or a combination of feature selection and predictive approaches was not widely available. Most of the machine learning algorithms used were common supervised algorithms (RF, SVM, KNN, CT, NB) (*n* = 98/174, 56.4%) (55). Still, the number of different methods used was 31 with one being a non-supervised algorithm (hierarchical clustering). There were five analyses (2 publications) that used an ensemble approach per our definition (46, 54). The top eight machine learning categories accounted for 95.3% (*n* = 166/174 classifiers) of the classifiers. This is likely because these are well-established methods that continue to perform well (Table 5; Supplementary Table S10). These are also amongst the most popular classification algorithms to use (56) and familiarity with given algorithms will result in greater use over unknown or emerging algorithms. The distribution of the usage of these methods remains consistent over time (Figure 6). The greatest change is observed in the usage of gradient boosting machines which has increased over the last several years. However, this method is not new as it was first introduced in 1999 (57), and other boosting methods were introduced prior to that, yet the reason for the increase in usage is unclear. A reason for the increase could be increased availability of or access to software that can reliably perform these algorithms. Examining the neural network usage over time, publications that were published more recently tended to describe their neural network in greater detail and there was a rise in the use of deep neural networks (Figure 8). Interesting and unique approaches to classifier validation were charted such as simulating an additional dataset and experimental validation. Twenty-one analyses used predictive probability to determine the most likely host, for three it was unclear, and the remainder did not appear to be using predictive probability to determine the most likely host or for other types of inferences.

A commonality in the publications themselves was an increase in complexity and specificity of host species in more recent publications with the advancement of data availability, computer science, and expansion of knowledge. Most of the publications originated from the United States, China, and the United Kingdom, however, this may reflect the English language limit implemented during the screening process. The earliest publication was published in 2008 and the most recent in 2022. Spikes in the number of publications were seen in 2010 and 2021. In 2010, all the publications used influenza virus genome data, and may be reflective of the 2009 H1N1 Pandemic with some publications specifically covering the 2009 H1N1 Pandemic (37, 38, 58, 59). Most publications from 2021 involved coronaviruses or a combination of coronaviruses, other viruses, and influenza viruses. This may be reflective of the SARS-CoV-2 pandemic originating in 2019 (27, 31, 60). At the analysis level, the same advancement can be seen with the significant increase in data availability and usage regarding both hosts and the number of sequences used. Another commonality was accuracy; measures most used were standard measures for machine learning classifiers such as accuracy, sensitivity, specificity, ROC, AUC, and *F*-score (*n* = 64/77 analyses used at least one of these accuracy measures, 83.1%). This may be useful for the comparison of results from various publications such as in a systematic review, however, the differences in materials and methods may make this an inappropriate comparison. Consistency in the method(s) of reporting accuracy is also important for a reader’s interpretability of results.

A limitation of this review was the heterogeneity of approaches which made it more difficult to succinctly chart all data. However, this was addressed by splitting data charting into three levels which were: publication information, analysis information, and classifier information. In this review, pathogens other than influenza viruses and coronaviruses were charted but the list of such pathogens likely does not completely represent this population of pathogens because the focus was on the two most important groups of pathogens affecting multiple species. A secondary limitation of this review was that data was not charted for the deployment methods used within the arithmetic design of the machine learning approaches in the analyses. It is recommended that future studies take this into account.

## 5 Conclusion

This scoping review aimed to address the research question “What machine learning methods have been applied to influenza virus and coronavirus genome data for identification of potential reservoirs?” Through conducting a scoping review, 53 publications were identified as relevant for this paper. It was found that 32 different machine learning algorithms were utilized, but the top eight algorithm classes accounted for 95.3 percent of the classifiers used. Additionally, it was identified that 41 different feature transformations were used, and 28 different types of feature selection were used as part of the machine learning analysis. The approaches used were heterogeneous and displayed the emerging nature of this research. This scoping review also allowed for the identification of several gaps in the current literature. For example, most publications used “avian” as a host group rather than specifying bird species. This is a gap in the current body of literature that should be addressed in future studies. Overall, the area of predictive modelling is developing fast, including novel methods. However, regardless of the methods used, the authors should consider the clinical, diagnostic, or surveillance question of interest, and sufficient specificity and quality of the data in combination with data analytics platforms for a thorough analysis.

## Data Availability

The original contributions presented in the study are included in the article/Supplementary material, further inquiries can be directed to the corresponding author.

## References

[1] Miller RS Sweeney SJ Slootmaker C Grear DA Di Salvo PA Kiser D. Cross-species transmission potential between wild pigs, livestock, poultry, wildlife, and humans: implications for disease risk Management in North America. Sci Rep. (2017) 7:7821. doi: 10.1038/s41598-017-07336-z 28798293 PMC5552697

[2] Parrish CR Holmes EC Morens DM Park E-C Burke DS Calisher CH. Cross-species virus transmission and the emergence of new epidemic diseases. Microbiol Mol Biol Rev. (2008) 72:457–70. doi: 10.1128/MMBR.00004-08 18772285 PMC2546865

[3] Claes F Kuznetsov D Liechti R Von Dobschuetz S Dinh Truong B Gleizes A. The EMPRES-i genetic module: a novel tool linking epidemiological outbreak information and genetic characteristics of influenza viruses. Database. (2014) 2014:bau008. doi: 10.1093/database/bau008 24608033 PMC3945526

[4] Haydon DT Cleaveland S Taylor LH Karen Laurenson M. Identifying reservoirs of infection: a conceptual and practical challenge. Emerg Infect Dis. (2002) 8:1468–73. doi: 10.3201/eid0812.010317, PMID: 12498665 PMC2738515

[5] Fermin G. Chapter 5 - Host Range, Host–Virus Interactions, and Virus Transmission. In: P Tennant G Fermin JE Foster, editors. Viruses [Internet]. London, United Kingdom: Academic Press (2018). p. 101–34. Available from: https://www.sciencedirect.com/science/article/pii/B978012811257100005X.

[6] Cassedy A Parle-McDermott A O’Kennedy R. Virus detection: a review of the current and emerging molecular and immunological methods. Front Mol Biosci. (2021) 8:637559. doi: 10.3389/fmolb.2021.637559 33959631 PMC8093571

[7] McLeish MJ Fraile A García-Arenal F. Evolution of plant–virus interactions: host range and virus emergence. Curr Opin Virol. (2019) 34:50–5. doi: 10.1016/j.coviro.2018.12.003 30654270

[8] Viana M Mancy R Biek R Cleaveland S Cross PC Lloyd-Smith JO. Assembling evidence for identifying reservoirs of infection. Trends Ecol Evol. (2014) 29:270–9. doi: 10.1016/j.tree.2014.03.002 24726345 PMC4007595

[9] Lee B Smith DK Guan Y. Alignment free sequence comparison methods and reservoir host prediction. Bioinformatics. (2021) 37:3337–42. doi: 10.1093/bioinformatics/btab338, PMID: 33964132 PMC8135978

[10] Ebrahimi M Aghagolzadeh P Shamabadi N Tahmasebi A Alsharifi M Adelson DL. Understanding the underlying mechanism of HA-subtyping in the level of physic-chemical characteristics of protein. PLoS One. (2014) 9:e96984. doi: 10.1371/journal.pone.0096984, PMID: 24809455 PMC4014573

[11] Moradi M Golmohammadi R Najafi A Moghaddam MM Fasihi-Ramandi M Mirnejad R. A contemporary review on the important role of in silico approaches for managing different aspects of COVID-19 crisis. Inform Med Unlocked. (2022) 28:100862. doi: 10.1016/j.imu.2022.100862, PMID: 35079621 PMC8776350

[12] Abd-Alrazaq A Alajlani M Alhuwail D Schneider J Al-Kuwari S Shah Z. Artificial intelligence in the fight against COVID-19: scoping review. J Med Internet Res. (2020) 22:e20756. doi: 10.2196/20756 33284779 PMC7744141

[13] Borkenhagen LK Allen MW Runstadler JA. Influenza virus genotype to phenotype predictions through machine learning: a systematic review. Emerg Microbes Infect. (2021) 10:1896–907. doi: 10.1080/22221751.2021.1978824 34498543 PMC8462836

[14] Tricco AC Lillie E Zarin W O’Brien KK Colquhoun H Levac D. PRISMA extension for scoping reviews (PRISMA-ScR): checklist and explanation. Ann Intern Med. (2018) 169:467–73. doi: 10.7326/M18-0850, PMID: 30178033

[15] Mendeley Ltd. Mendeley Desktop [Software]. Version 1.19.8. London: Mendeley Ltd. (2020).

[16] Grange ZL Goldstein T Johnson CK Anthony S Gilardi K Daszak P. Ranking the risk of animal-to-human spillover for newly discovered viruses. Proc Natl Acad Sci USA. (2021) 118. doi: 10.1073/pnas.2002324118, PMID: 33822740 PMC8053939

[17] Clarivate. EndNote [Software]. Version 20. Philadelphia, PA: Clarivate (2020).

[18] R Core Team. R: A language and environment for statistical computing. Vienna: R Foundation for Statistical Computing (2023) Available at: https://www.R-project.org/.

[19] RStudio Team. RStudio. Boston: PBC (2022) Available at: http://www.rstudio.com/.

[20] Wickham H Averick M Bryan J Chang W McGowan L François R. Welcome to the Tidyverse. J Open Source Softw. (2019) 4:1686. doi: 10.21105/joss.01686

[21] Csardi G Nepusz T. The Igraph software package for complex network research. InterJournal Complex Syst. (2006) 1695:1–9. Available at: https://igraph.org

[22] Bartoszewicz JM Seidel A Renard BY. Interpretable detection of novel human viruses from genome sequencing data. NAR Genom Bioinform. (2021) 3:lqab004. doi: 10.1093/nargab/lqab004, PMID: 33554119 PMC7849996

[23] Sutanto K Turcotte M. (2021). Extracting and evaluating features from RNA virus sequences to predict host species susceptibility using deep learning. 2021 13th International Conference on Bioinformatics and Biomedical Technology. 81–89. New York, NY: ACM.

[24] Aguas R Ferguson NM. Feature selection methods for identifying genetic determinants of host species in RNA viruses. PLoS Comput Biol. (2013) 9:e1003254. doi: 10.1371/journal.pcbi.1003254, PMID: 24130470 PMC3794897

[25] Babayan SA Orton RJ Streicker DG. Predicting reservoir hosts and arthropod vectors from evolutionary signatures in RNA virus genomes. Science. (2018) 362:577–80. doi: 10.1126/science.aap9072 30385576 PMC6536379

[26] Bergner LM Mollentze N Orton RJ Tello C Broos A Biek R. Characterizing and evaluating the zoonotic potential of novel viruses discovered in vampire bats. Viruses. (2021) 13:252. doi: 10.3390/v13020252 33562073 PMC7914986

[27] Guo Q Li M Wang C Guo J Jiang X Tan J. Predicting hosts based on early SARS-CoV-2 samples and analyzing the 2020 pandemic. Sci Rep. (2021) 11:17422. doi: 10.1038/s41598-021-96903-6, PMID: 34465838 PMC8408148

[28] Li H Sun F. Comparative studies of alignment, alignment-free and SVM based approaches for predicting the hosts of viruses based on viral sequences. Sci Rep. (2018) 8:10032. doi: 10.1038/s41598-018-28308-x 29968780 PMC6030160

[29] Mock F Viehweger A Barth E Marz M. VIDHOP, viral host prediction with deep learning. Bioinformatics. (2021) 37:318–25. doi: 10.1093/bioinformatics/btaa705 32777818 PMC7454304

[30] Mollentze N Babayan SA Streicker DG. Identifying and prioritizing potential human-infecting viruses from their genome sequences. PLoS Biol. (2021) 19:e3001390. doi: 10.1371/journal.pbio.3001390, PMID: 34582436 PMC8478193

[31] Yang Y Guo J Wang P Wang Y Minghao Y Wang X. Reservoir hosts prediction for COVID-19 by hybrid transfer learning model. J Biomed Inform. (2021) 117:103736. doi: 10.1016/j.jbi.2021.103736, PMID: 33711547 PMC7942058

[32] Young F Rogers S Robertson DL. Predicting host taxonomic information from viral genomes: a comparison of feature representations. PLoS Comput Biol. (2020) 16:e1007894. doi: 10.1371/journal.pcbi.1007894 32453718 PMC7307784

[33] Zhang Z Cai Z Tan Z Congyu L Jiang T Zhang G. Rapid identification of human-infecting viruses. Transbound Emerg Dis. (2019) 66:2517–22. doi: 10.1111/tbed.13314, PMID: 31373773 PMC7168554

[34] Davis P Russell JA. (2021). A genotype-to-phenotype modeling framework to predict human pathogenicity of novel coronaviruses. *BioRxiv*. Available at: 10.1101/2021.09.18.460926. [Epub ahead of preprint]

[35] Kuzmin K Adeniyi AE DaSouza AK Lim D Nguyen H Molina NR. Machine learning methods accurately predict host specificity of coronaviruses based on spike sequences alone. Biochem Biophys Res Commun. (2020) 533:553–8. doi: 10.1016/j.bbrc.2020.09.010, PMID: 32981683 PMC7500881

[36] Yerukala Sathipati S Shukla SK Ho SY. Tracking the amino acid changes of spike proteins across diverse host species of severe acute respiratory syndrome coronavirus 2. iScience. (2022) 25:103560. doi: 10.1016/j.isci.2021.103560, PMID: 34877480 PMC8638202

[37] Attaluri PK Zheng X Chen Z Lu G. (2009). Applying machine learning techniques to classify H1N1 viral strains occurring in 2009 flu pandemic. Available at: https://www.researchgate.net/publication/253864701_Applying_machine_learning_techniques_to_classify_H1N1_viral_strains_occurring_in_2009_flu_pandemic

[38] Meroz D Yoon S-W Ducatez MF Fabrizio TP Webby RJ Hertz T. Putative amino acid determinants of the emergence of the 2009 influenza A (H1N1) virus in the human population. Proc Natl Acad Sci USA. (2011) 108:13522–7. doi: 10.1073/pnas.1014854108 21808039 PMC3158228

[39] Eng CLP Tong J Tan T. Predicting zoonotic risk of influenza A viruses from host tropism protein signature using random forest. Int J Mol Sci. (2017) 18:1135. doi: 10.3390/ijms18061135 28587080 PMC5485959

[40] Qiang X Kou Z. Prediction of interspecies transmission for avian influenza A virus based on a back-propagation neural network. Math Comput Model. (2010) 52:2060–5. doi: 10.1016/j.mcm.2010.06.008

[41] Scarafoni D Telfer BA Ricke DO Thornton JR Comolli J. Predicting influenza A tropism with end-to-end learning of deep networks. Health Secur. (2019) 17:468–76. doi: 10.1089/hs.2019.0055 31859569

[42] Tang Q Song Y Shi M Cheng Y Zhang W Xia X-Q. Inferring the hosts of coronavirus using dual statistical models based on nucleotide composition. Sci Rep. (2015) 5:17155. doi: 10.1038/srep17155 26607834 PMC4660426

[43] Wardeh M Baylis M Blagrove MSC. Predicting mammalian hosts in which novel coronaviruses can be generated. Nat Commun. (2021) 12:780. doi: 10.1038/s41467-021-21034-5, PMID: 33594041 PMC7887240

[44] Gaunt ER Digard P. Compositional biases in RNA viruses: causes, consequences and applications. WIREs RNA. (2022) 13:e1679. doi: 10.1002/wrna.1679, PMID: 34155814 PMC8420353

[45] Xu Y Wojtczak D. (2021). Predicting influenza A viral host using PSSM and word embeddings. 2021 IEEE Conference on Computational Intelligence in Bioinformatics and Computational Biology (CIBCB). 1–10

[46] Kou Z Li J Fan X Kosari S Qiang X. Predicting Cross-species infection of swine influenza virus with representation learning of amino acid features. Comput Math Methods Med. (2021) 2021:1–12. doi: 10.1155/2021/6985008, PMID: 34671417 PMC8523279

[47] Eng CLP Tong JC Tan TW. Predicting host tropism of influenza A virus proteins using random forest. BMC Med Genet. (2014) 7:S1. doi: 10.1186/1755-8794-7-S3-S1 PMC4290784 25521718

[48] Kwon E Cho M Kim H Son HS. A study on host tropism determinants of influenza virus using machine learning. Curr Bioinform. (2020) 15:121–34. doi: 10.2174/1574893614666191104160927

[49] Shaltout NA El-Hefnawi M Rafea A Moustafa A. (2014). Information gain as a feature selection method for the efficient classification of influenza based on viral hosts. Proceedings of the World Congress on Engineering. Available at: https://www.researchgate.net/publication/286743906_Information_Gain_as_a_Feature_Selection_Method_for_the_Efficient_Classification_of_Influenza_Based_on_Viral_Hosts

[50] Shaltout N Rafea A Moustafa A El-Hefnawi M. Using information gain to compare the Efficiency of machine learning techniques when classifying influenza based on viral hosts In: Transactions on engineering technologies. Dordrecht: Springer (2015). 707–22.

[51] Cai J Luo J Wang S Yang S. Feature selection in machine learning: a new perspective. Neurocomputing. (2018) 300:70–9. doi: 10.1016/J.NEUCOM.2017.11.077

[52] Eng CLP Tong JC Tan TW. Distinct host tropism protein signatures to identify possible zoonotic influenza A viruses. PLoS One. (2016) 11:e0150173. doi: 10.1371/journal.pone.0150173 26915079 PMC4767729

[53] Kou Z Lei FM Wang SY Zhou YH Li TX. Molecular patterns of avian influenza A viruses. Chin Sci Bull. (2008) 53:2002–7. doi: 10.1007/s11434-008-0236-2

[54] Kargarfard F Sami A Mohammadi-Dehcheshmeh M Ebrahimie E. Novel approach for identification of influenza virus host range and zoonotic transmissible sequences by determination of host-related associative positions in viral genome segments. BMC Genomics. (2016) 17:925. doi: 10.1186/s12864-016-3250-9 27852224 PMC5112743

[55] Hamilton AJ Strauss AT Martinez DA Hinson JS Levin S Lin G. Machine learning and artificial intelligence: applications in healthcare epidemiology. Antimicrob Steward Healthc Epidemiol. (2021) 1:e28. doi: 10.1017/ash.2021.192 36168500 PMC9495400

[56] Sarker IH. Machine learning: algorithms, real-world applications and research directions. SN Comput Sci. (2021) 2:160. doi: 10.1007/s42979-021-00592-x, PMID: 33778771 PMC7983091

[57] Friedman JH. Greedy function approximation: a gradient boosting machine. Ann Stat. (2001) 29:1189–232. doi: 10.1214/aos/1013203451

[58] Cook PW Stark T Jones J Kondor R Zanders N Benfer J. Detection and characterization of swine origin influenza A(H1N1) pandemic 2009 viruses in humans following zoonotic transmission. J Virol. (2020) 95:e01066. doi: 10.1128/JVI.01066-20 33115872 PMC7944445

[59] Hu W. Novel host markers in the 2009 pandemic H1N1 influenza A virus. J Biomed Sci Eng. (2010) 3:584–601. doi: 10.4236/jbise.2010.36081

[60] Guo Q Li M Wang C Fang Z Wang P Tan J. (2020). Host and infectivity prediction of Wuhan 2019 novel coronavirus using deep learning algorithm. *BioRxiv*: Available at: 10.1101/2020.01.21.914044. [Epub ahead of preprint].

