# Supplementary material for: Predicting host species susceptibility to influenza viruses and coronaviruses using genome data and machine learning: a scoping review
Source: Front Vet Sci. 2024 Sep 25;11:1358028. doi: 10.3389/fvets.2024.1358028 (PMC11462629; doi:10.3389/fvets.2024.1358028)
Supplement: Supplementary file 1 [file Table_1.DOCX]

S1 Table: The search strings used during database search for the scoping review.

| **Clarivate, Web of Science Core Collection – Date Range 2000-2022** |
| --- |
| ((TS=(zoono* or between-species transmission or host range or cross-species transmission or pathogen spillover or spillover or host tropism or host specificity or reservoir )) AND TS=(machine learning or big data or convolution neural network or deep learning or network analysis or bioinformatics or predictive model* or unsupervised learning or supervised learning or semi-supervised learning or active learning or algorithm or ai or artificial intelligence)) AND TS=(influenza* or Orthomyxovir* or flu or Coronavir* or covid or IAV ) |
| **Elsevier, Engineering Village- Inspec and Compendex** |
| ((( ((((((({learning (artificial intelligence)} OR {neural networks} OR {genetic algorithms} OR {artificial intelligence} OR {semi-supervised learning} OR {deep learning} OR {convolutional neural networks}) WN CV)) AND (2000-2022 WN YR)) OR ((($machine $learning OR $big $data OR $convolution $neural $network OR $deep $learning OR $network $analysis OR $bioinformatics OR $predictive model* OR $unsupervised $learning OR $supervised $learning OR $semi-supervised $learning OR $active $learning OR $algorithm OR $ai OR $artificial $intelligence) WN KY) AND (2000-2022 WN YR)))) AND (((zoono* OR $between-species $transmission OR $host $range OR $cross-species $transmission OR $pathogen $spillover OR $spillover OR $host $tropism OR $host $specificity OR $reservoir) WN KY) AND (2000-2022 WN YR)) AND ((((influenza* OR Orthomyxovir* OR $flu OR Coronavir* OR $covid OR $IAV)) WN KY) AND (2000-2022 WN YR)))))) |
| **National Center for Biotechnology Information (NCBI), PubMed – Date Range 2000-2022** |
| (((((((Artificial Intelligence [mh]) OR (Computational Biology[mh])) OR (Neural Networks, Computer [mh])) OR (Big Data[mh:noexp])) OR (algorithms [mh:noexp])) OR (machine learning[Text Word] OR big data[Text Word] OR convolution neural network[Text Word] OR deep learning[Text Word] OR network analysis[Text Word] OR bioinformatics[Text Word] OR predictive model*[Text Word] OR unsupervised learning[Text Word] OR supervised learning[Text Word] OR semi-supervised learning[Text Word] OR active learning[Text Word] OR algorithm[Text Word] OR ai[Text Word] OR artificial intelligence[Text Word])) AND ((zoono*[Text Word] OR between-species transmission[Text Word] OR host range[Text Word] OR cross-species transmission[Text Word] OR pathogen spillover[Text Word] OR spillover[Text Word] OR host tropism[Text Word] OR host specificity[Text Word] OR reservoir[Text Word]) OR ((((((Host Specificity [mh:noexp]) OR (zoonoses [mh:noexp]) OR viral (zoonoses[mh:noexp])) OR (Viral Tropism [mh:noexp])) OR (disease reservoirs[mh:noexp])) OR (disease vectors[mh:noexp])) OR (disease transmission, infectious[mh])))) AND ((influenza*[Text Word] OR Orthomyxovir*[Text Word] OR flu[Text Word] OR Coronavir*[Text Word] OR covid[Text Word] OR IAV[Text Word]) OR (((((Influenza, Human [mh:noexp]) OR (Orthomyxoviridae [mh])) OR (Coronaviridae [mh])) OR (COVID-19 [mh])) OR (Coronaviridae Infections [mh]))) |
| **Ovid Technologies Inc., MEDLINE** |
| \| **#** \|  \| **Query** \| \| --- \| --- \| --- \| \| 1 \|  \| Influenza, Human/ \| \| 2 \|  \| exp Orthomyxoviridae/ \| \| 3 \|  \| exp Coronaviridae/ \| \| 4 \|  \| exp COVID-19/ \| \| 5 \|  \| exp Coronaviridae Infections/ \| \| 6 \|  \| (influenza* or Orthomyxovir* or flu or Coronavir* or covid or IAV).tw. \| \| 7 \|  \| 1 or 2 or 3 or 4 or 5 or 6 \| \| 8 \|  \| Host Specificity/ \| \| 9 \|  \| zoonoses/ or viral zoonoses/ \| \| 10 \|  \| Viral Tropism/ \| \| 11 \|  \| exp disease transmission, infectious/ or disease reservoirs/ or disease vectors/ \| \| 12 \|  \| (zoono* or between-species transmission or host range or cross-species transmission or pathogen spillover or spillover or host tropism or host specificity or reservoir).tw. \| \| 13 \|  \| 8 or 9 or 10 or 11 or 12 \| \| 14 \|  \| exp Artificial Intelligence/ \| \| 15 \|  \| exp Computational Biology/ \| \| 16 \|  \| exp Neural Networks, Computer/ \| \| 17 \|  \| Big Data/ \| \| 18 \|  \| algorithms/ \| \| 19 \|  \| (machine learning or big data or convolution neural network or deep learning or network analysis or bioinformatics or predictive model* or unsupervised learning or supervised learning or semi-supervised learning or active learning or algorithm or ai or artificial intelligence).tw. \| \| 20 \|  \| 14 or 15 or 16 or 17 or 18 or 19 \| \| 21 \|  \| 7 and 13 and 20 \| \| 22 \|  \| limit 21 to yr="2000 - 2023" \| \|  \|  \|  \| |
| **ProQuest, Coronavirus Research Database – Date Range 2000-2022** |
| ab((influenza* or Orthomyxovir* or flu or Coronavir* or covid or IAV) AND (zoono* or between-species transmission or host range or cross-species transmission or pathogen spillover or spillover or host tropism or host specificity or reservoir) AND (machine learning or big data or convolution neural network or deep learning or network analysis or bioinformatics or predictive model* or unsupervised learning or supervised learning or semi-supervised learning or active learning or algorithm or ai or artificial intelligence)) OR ti((influenza* or Orthomyxovir* or flu or Coronavir* or covid or IAV) AND (zoono* or between-species transmission or host range or cross-species transmission or pathogen spillover or spillover or host tropism or host specificity or reservoir) AND (machine learning or big data or convolution neural network or deep learning or network analysis or bioinformatics or predictive model* or unsupervised learning or supervised learning or semi-supervised learning or active learning or algorithm or ai or artificial intelligence)) |
