# Supplementary material for: Predicting host species susceptibility to influenza viruses and coronaviruses using genome data and machine learning: a scoping review
Source: Front Vet Sci. 2024 Sep 25;11:1358028. doi: 10.3389/fvets.2024.1358028 (PMC11462629; doi:10.3389/fvets.2024.1358028)
Supplement: Supplementary file 2 [file Table_2.DOCX]

S2 Table: Forms used for screening and data charting.

| **Level 1 – Title/Abstract Screening** | | |
| --- | --- | --- |
| Question | Answers | Effect |
| 1. Is the abstract present in the meta-data? | Yes | Continue to Q2 |
|  | No | Add metadata continue to Q2 (Q1 in Level 1B) |
| 2. Is the title/abstract available in English? | Yes | Continue to Q3 |
|  | No | Exclude |
|  | Unclear | Continue to Q3 |
| 3. Does the abstract/title include machine learning or predictive classifierling with regard to genome data (bioinformatics)? | Yes | Continue to Q4 |
|  | No | Exclude |
|  | Unclear | Continue to Q4 |
| 4. Does the abstract/title include host-range or between-species transmission potential? | Yes | Continue to Q5 |
|  | No | Exclude |
|  | Unclear | Continue to Q5 |
| 5. Does the abstract/title include influenza virus or coronaviruses? | Yes | Continue to Q6 |
|  | No | Exclude |
|  | Unclear | Continue to Q6 |
| 6. Is the citation primary research? | Yes | Include in Level 2 |
|  | No | Continue to Q7 |
|  | Unclear | Include in Level 2 |
| 7. Is the publication a review? | Yes | Exclude, flag as review |
|  | No | Exclude |

| **Level 2- Full-Text Screening** | | |
| --- | --- | --- |
| Question | Answer | Effect |
| 1. Is the full body text (beyond title/abstract) available in English? | Yes | Continue to Q2 |
|  | No | Exclude |
|  | Unclear | Continue to Q2 |
| 2. Does the full text include machine learning as predictive classifierling with regard to genome data? | Yes | Continue to Q3 |
|  | No | Exclude |
|  | Unclear | Continue to Q3 |
| 3. Does the full text include using a classifier for host-range or between-species transmission potential? | Yes | Continue to Q4 |
|  | No | Exclude |
|  | Unclear | Continue to Q4 |
| 4. Does the full text include influenza virus or coronaviruses? | Yes | Continue to Q5 |
|  | No | Exclude |
|  | Unclear | Continue to Q5 |
| 5. Is the citation primary research? | Yes | Include |
|  | No | Continue to Q6 |
|  | Unclear | Include |
| 6. Is the publication a review? | Yes | Exclude, flag as review |
|  | No | Exclude |

| **Data Charting: Publication Information** | | |
| --- | --- | --- |
| Question | Answer | Effect |
| 1. Ref ID | The number provided by Distiller |  |
| 2. Year of Publication | YYYY (ex. 2021) |  |
| 3. What is the first author affiliation? | Select all that apply. If not stated select "Not Stated". If the answer was not present add it to list. |  |
| 4. What country is the first author affiliated with | Select all that apply. If not stated select "Not Stated". If the answer was not present add it to list. |  |
| 5. What department is the first author affiliated with? | Select all that apply. If not stated select "Not Stated". If the answer was not present add it to list. |  |
| 6. What is the publication type? | Journal Article |  |
|  | Conference Proceeding |  |
|  | Other |  |
| 7. What are the objectives of the publication? | Copy and paste directly from the introduction or if the introduction is not present the abstract. If an objective(s) is not clearly stated, summarize it. |  |
| 8. How many analyses were performed? | Type the number. Where analysis is defined as a set where one or more classification classifiers use the same training and test source population in one or more input formats. | Create an equal number of analysis information forms. |

| **Data Charting: Analysis Information** | | |
| --- | --- | --- |
| Question | Answer | Effect |
| 1. Which analysis is this? | State number in terms of order presented in the publication. |  |
| 2. What viruses were used? (select all that apply) | Coronavirus | Q3, Q4, Q5, and Q7 become available to be answered. |
|  | Influenza | Q8, Q9, Q10, and Q12 become available to be answered. |
|  | Other: Fill in the text | Q13 and Q14 become available to be answered. |
|  | Not Stated | Q16 is the next question. |
| *Coronavirus Information* | | |
| 3. Which coronaviruses were used? | Select all that apply. If not stated select "Not Stated". If the answer was not present add it to list. Answer to the most specific level that is stated. |  |
| 4. Which database were the coronavirus sequences retrieved from? | Select all that apply. If not stated select "Not Stated". If the answer was not present add it to list. |  |
| 5. What taxonomic level(s) were hosts defined at and how many groups were there used as input into the analysis? | Select all that apply and provide the number within the selected group. If not stated select "Not Stated". Groups include Domain, Kingdom, Phylum, Class, Order, Suborder, Family, Genus, and Species. | If Species is selected Q6 becomes available to be answered. |
| 6. What were the species used? | Select all that apply and provide the total number of species. If not stated select "Not Stated". If the answer was not present add it to list. |  |
| 7. What type of sequence was used? | Select all that apply. If not stated select "Not Stated". If the answer was not present add it to list. |  |
| *Influenza Virus Information* | | |
| 8. Which influenza viruses were used? | Select all that apply. If not stated select "Not Stated". If the answer was not present add it to the list. |  |
| 9. Which database were the influenza sequences retrieved from? | Select all that apply. If not stated select "Not Stated". If the answer was not present add it to the list. |  |
| 10. What taxonomic level(s) were hosts defined at and how many groups were there used as input into the analysis? | Select all that apply and provide the number within the selected group. If not stated select "Not Stated". Groups include Domain, Kingdom, Phylum, Class, Order, Suborder, Family, Genus, and Species. | If Species is selected Q11 becomes available to be answered. |
| 11. What were the species used? | Select all that apply and provide the total number of species. If not stated select "Not Stated". If the answer was not present add it to list. |  |
| 12. What type of sequence was used? | Select all that apply. If not stated select "Not Stated". Options include PB2, PB1, HA, NA, NP, NA, M, and NS. |  |
| *Other Viruses* | | |
| 13. What level(s) were other viruses identified at? | Select all that apply. If not stated select "Not Stated". If the answer was not present add it to list. |  |
| 14. What taxonomic level(s) were hosts defined at and how many groups were there used as input into the analysis? | Select all that apply and provide the number within the selected group. If not stated select "Not Stated". Groups include Domain, Kingdom, Phylum, Class, Order, Suborder, Family, Genus, and Species. | If Species is selected Q15 becomes available to be answered. |
| 15. What were the species used? | Select all that apply and provide the total number of species. If not stated select "Not Stated". If the answer was not present add it to list. |  |
| *Host Genetics* | | |
| 16. Was genetic data from the host(s) used? | Yes |  |
|  | No |  |
|  | Unclear |  |
|  | Other- Fill in the text. |  |
| *Data Processing* | | |
| 17. What input structure were sequences used in? | Select all that apply. If not stated select "Not Stated". If the answer was not present add it to list. |  |
| 18. What other analysis was used on the sequences? | Select all that apply. If not stated select "Not Stated". If the answer was not present add it to list. |  |
| 19. What were the selection criteria for the sequences used? | Copy and paste or summarize all steps used to get from the number of sequences available to the number of sequences used in the classifier(s). |  |
| *Classifier* | | |
| 20. What programming language and/or software was used? | Select all that apply. If not stated select "Not Stated". If the answer was not present add it to list. |  |
| 21. How many machine learning classifiers were built? | Numeric Answer > 0. | Corresponds to the number of sub-forms that were filled in for Q22. |
| 22. Classifier information subform | See Subform 1: Classifier Information | An additional sub-form was completed for the number of classifiers identified in Q21. |
| *Data Outcomes* | | |
| 23. What measures were used to evaluate the classifiers (ex. accuracy, etc.)? | Select all that apply. If not stated select "Not Stated". If the answer was not present add it to list. |  |
| 24. Was the accuracy/predictive probability of the curve used for the determination of hosts? | Yes |  |
|  | No |  |
|  | Unclear |  |
| 25. What were the visual displays showing? | Select all that apply. If no visuals, select "No Visuals". If the answer was not present add it to list. |  |
| 26. Additional Comments | Type any additional comments regarding the current analysis. |  |

| **Data Charting: Subform 1: Classifier Information** | | |
| --- | --- | --- |
| 1. Which classifier is this? | Enter a number based on the order presented in the publication. (starting at one with each new analysis). |  |
| 2. Has this classifier been used previously? | Yes | Provide the reference in which the classifier was previously used. |
|  | No |  |
|  | Unclear |  |
| 3. What feature selection classifier was used? | Select all that apply. If not stated select "Not Stated". If not used select "Not used". If the answer was not present add it to list. |  |
| 4. What class of machine learning was used? (select all that apply) | Supervised | Q5 becomes available to answer. |
|  | Semi-Supervised | Q6 becomes available to answer. |
|  | Unsupervised | Q7 becomes available to answer. |
|  | Not Stated | Answer Q8 next. |
| 5. What supervised classifier was used? | Select all that apply. If not stated select "Not Stated". If not used select "Not used". If the answer was not present add it to list. |  |
| 6. What semi-supervised classifier was used? | Select all that apply. If not stated select "Not Stated". If not used select "Not used". If the answer was not present add it to the list. |  |
| 7. What unsupervised classifier was used? | Select all that apply. If not stated select "Not Stated". If not used select "Not used". If the answer was not present add it to list. |  |
| 8. How many sequences were used to test and train this classifier? | Numeric answer. If not stated leave blank.  Calculate the total amount of sequences used for one classifier (summation of all hosts) or in cases where the influenza segments are utilized calculate the average per segment. |  |
| 9. What type of validation was used? | Select all that apply. If not stated select "Not Stated". If not used select "Not used". If the answer was not present add it to list. |  |
| 10. Were multiple hosts used to train the classifier? | Yes |  |
|  | No |  |
|  | Unclear |  |
| 11. Were multiple viral groups used to train the classifier? | Yes |  |
|  | No |  |
|  | Unclear |  |
| 12. Was the classifier used for the prediction of the host(s) or prediction of a spillover event? (Select all that apply) | Prediction of Host |  |
|  | Prediction of Spillover |  |
|  | Not Stated |  |
|  | Other | Type what other means. |
| Was this classifier identified as the best-performing classifier? (Within the given analysis) | Yes  This was determined based on what the author deemed to be the best classifier or if not explicitly stated the classifier with the highest measure of accuracy. |  |
|  | No |  |
|  | Not Stated  Select if not stated or if only one classifier was used within an analysis. |  |
