# Supplementary material for: Predicting host species susceptibility to influenza viruses and coronaviruses using genome data and machine learning: a scoping review
Source: Front Vet Sci. 2024 Sep 25;11:1358028. doi: 10.3389/fvets.2024.1358028 (PMC11462629; doi:10.3389/fvets.2024.1358028)
Supplement: Supplementary file 3 [file Table_3.DOCX]

Table S3: Funding Sources for publications included in data charting.

| **Reference** | **Funding*** |
| --- | --- |
|  |  |
| Aguas & Ferguson (2013) | We thank the EU FP7 EMPERIE project, the NIGMS MIDAS programme, and the MRC for research funding. |
| Ali et al. (2022) | S.A. was supported by a Georgia State University (GSU) Molecular Basis of Disease (MBD) fellowship, B.B. was supported by the Chemistry department at GSU, and M.P. was supported by a GSU/ department of Computer Science start-up grant. |
| Attaluri, Zheng, Chen & Lu (2009) | This publication was made possible by NIH grant numbers P20 RR016469 from the INBRE Program of the National Center for Research Resources and R01 LM009985-01A1. n. The authors also, acknowledge the UCRCA, the University of Nebraska at Omaha, for continuous funding support. |
| Attaluri, Chen, & Lu (2010) | This publication was made possible by NIH grant numbers P20 RR016469 and R01 LM009985-01A1. The authors also acknowledge the University Committee on Research and Creative Activity (UCRCA), University of Nebraska at Omaha, for continuous funding support. |
| Babayan, Orton, & Streicker (2018) | Funding: S.A.B. was supported by a Glasgow University Research Fellowship and the BBSRC (BB/M012956/1). D.G.S. was supported by a Sir Henry Dale Fellowship, jointly funded by the Wellcome Trust and Royal Society (102507/Z/13/Z). Additional funding was provided from the Medical Research Council (MC_UU_12014/12). |
| Bartoszewicz, Seidel, & Renard (2021) | German Academic Scholarship Foundation (to J.M.B.); Federal Ministry of Education and Research (BMBF) Computational Life Sciences Initiative (project DeepPath, to B.Y.R.); the BMBF-funded de.NBI Cloud within the German Network for Bioinformatics Infrastructure (de.NBI) [031A537B, 031A533A, 031A538A, 031A533B, 031A535A, 031A537C, 031A534A, 031A532B]. |
| Bergner et al. (2021) | Funding: Funding was provided by theWellcome Trust (Wellcome-Beit Prize: 102507/Z/13/A;Wellcome Senior Research Fellowship: 217221/Z/19/Z). L.M.B. was funded by the Human Frontier Science Program (Grant: RGP0013/2018), and R.J.O. was funded by the MRC (Grant: MC_UU_12014/12). |
| Brierley et al. (2021) | LB acknowledges funding from a Medical Research Council Skills Development Fellowship award, grant number MR/T027355/1, https://mrc. ukri.org/. |
| Chrysostomou, Alexandrou, Nicolaou, & Seker (2021) | No Funding Source Stated. |
| Cook et al. (2020) | P.W.C. was supported by the Association of Public Health Laboratories. N.Z. was supported by Chickasaw Nation Industries. |
| Davis & Russell (2021) | No Funding Source Stated. |
| Dhanoa, Manicassamy, & Chattopadhyay (2018) | No Funding Source Stated. |
| ElHefnawi & Sherif (2014) | No Funding Source Stated. |
| Eng, Tong & Tan (2014) | The work was supported by Research Scholarship awarded to CLPE from the National University of Singapore. Funding for publication of this article was provided by the Department of Biochemistry, National University of Singapore. |
| Eng, Tong, & Tan (2016) | The authors have no support or funding to report. |
| Eng, Tong, & Tan (2017) | The work was supported by the National University of Singapore Research Scholarship awarded to Christine L. P. Eng from the National University of Singapore. |
| Guo et al. (2020) | The National Key Research and Development Program of China (2017YFC1200205), the National Natural Science Foundation of China (31671366 and 91231119), and the Special Research Project of ‘Clinical Medicine + X’ by Peking University. |
| Guo et al. (2021) | This article was funded by National Key Research and Development Program of China (Grant no.  2017YFC1200205), National Natural Science Foundation of China (Grant no. 32070667) and High Performance Computing Platform of the Center for Life Science of Peking University. |
| Hu (2010a) | We thank Houghton College for its financial support. |
| Hu (2010b) | We thank Houghton College for its financial support. |
| Hu (2010c) | We thank Houghton College for its financial support. |
| Kargarfard, Sami, Mohammadi-Dehcheshmeh, & Ebrahimie (2016) | No Funding Source Stated. |
| Kincaid (2018) | No Funding Source Stated. |
| King, Miller, Jones, & Hu (2010) | We would like to thank the Summer Research Institute at Houghton College for the funding of this study. |
| Kou et al. (2008) | Supported by the Special Basic Research Funds of China (Grant No. 2007FY210700), Major Program of National Natural Science Foundation of China (Grant No. 90608020), the National Basic Research Program of China (Grant Nos. 2005CB523007 and 2005CB523004), Research Fund for the Doctoral Program of Higher Education of China (Grant No. 20050487037), the Knowledge Innovation Project of the Chinese Academy of Sciences (Grant No. KSCX2-YW-N-065) and the Sixth Framework Program in European (Grant Nos. SP5B-CT-2006-044161 and SP5B-CT-2006- 044405). |
| Kou, Li, Fan, Kosari, & Qiang (2021) | This work was supported by the National Natural Science Foundation of China (61972109, 62172114). |
| Kuzmin et al. (2020) | This research was supported in part by a Georgia State University Molecular Basis of Disease fellowship (KK). |
| Kwon, Cho, Kim, & Son (2020) | This work was supported by grants from the National Research Foundation of Korea funded by the Ministry of Education (No. 2017R1D1A1B03033413) and the Ministry of Science and ICT (No. 2019R1H1A1085901). |
| Lee, Smith, & Guan (2021) | Financial Support: none declared. |
| H. Li & Sun (2018) | This research is supported by the US National Science Foundation (NSF) [DMS-1518001] and National Institutes of Health (NIH) [R01GM120624]. |
| J. Li et al. (2020) | This work was supported by grants from the National Science and Technology Major Project of China (2017ZX10305501, 2018ZX10732401, 2018ZX10711001-003, AWS16J020, and BWS16J008), the Key Project of China State Key Laboratory of Pathogen and Biosecurity of China (SKLPBS1806 and SKLPBS1407), the National Natural Science Foundation of China (31200119), and the National Key Research and Development Program of China (2018YFC1200100). |
| Meroz et al. (2011) | This work was supported by the Specific Targeted Research Project project “EuroFlu,” funded by the FP6 European Commission Program (SP5B-CT-2007-044098 to N.B.-T.) R.W. was supported in part by the National Institute of Allergy and Infectious Diseases, National Institutes of Health, Department of Health and Human Services, under Contract HHSN266200700005C. D.M. was supported by the Edmond J. Safra Bioinformatics program at Tel Aviv University. |
| Mock, Viehweger, Barth, & Marz (2021) | This work was supported by the German Research Foundation (DFG) [CRC 1076] ‘AquaDiva’, subproject A06 (M.M.); and DFG [SPP 1596] MA 5082/ 7-1 (M.M.). |
| Mollentze, Babayan, & Streicker (2021) | D.G.S. and N.M. were supported by a Wellcome Senior Research Fellowship (217221/Z/19/Z). Additional funding was provided by the Medical Research Council through program grants MC_UU_12014/8 and MC_UU_12014/12. |
| Qiang & Kou (2019) | This research was funded by the Chinese National Natural Science Foundation (61379059, 61472372, 61632002). |
| Qiang & Kou (2010) | The work was supported by National Natural Science Foundation of China (30900060, 60974112, 60971085, 60910002), 863 Program of China (2009AA012413). |
| Sathipati, Shukla, & Ho (2022) | This work was supported in part by the Marshfield Clinic Research Institute, Marshfield, WI. |
| Scarafoni, Telfer, Ricke, Thornton, & Comolli (2019) | This material is based upon work supported under Air Force Contract No. FA8721-05-C-0002 and/or FA8702-15-D-0001. |
| Shaltout, El-Hefnawi, Rafea, & Moustafa (2014) | This work was supported in part by Department of Computer Science in the American University of Cairo. |
| Shaltout, Rafea, Moustafa, & ElHefnawi (2015) | No Funding Source Stated. |
| Sherif, Zayed, & Fakhr (2017) | No Funding Source Stated. |
| Sun et al. (2021) | This work was supported by grants from the National Natural Science Foundation of China (3191101787, 31970153, 31630079), the National Key R&D Program of China (2016YFD0500206), the Strategic Priority Research Program of the Chinese Academy of Sciences (XDB29010000) and the Mega-Project of Guangxi Natural Science Foundation (2015GXNSFEA139002). J.L. is supported by Youth Innovation Promotion Association of CAS (2019091). |
| Sutanto & Turcotte (2021) | This study was supported by funding from Natural Sciences and Engineering Research Council of Canada, and computing facilities provided by Compute Ontario and Compute Canada. |
| Tang et al. (2015) | This work was supported by the 100-talent program grant from the Chinese Academy of Sciences. |
| Wang, Ma, Kou, Zhou, & Liu (2013) | This work is supported by the National Natural Science Foundation of China (90608020 and 30971642), the Program for New Century Excellent Talents in University (NCET-060651) and the Natural Science Foundation of Hubei Province of China (2009CDA161). |
| Wang, Kou, Duan, Ma, & Zhou (2013) | This work is supported by the National Natural Science Foundation of China (90608020 and 30971642), the Program for New Century Excellent Talents in University (NCET-060651) and the Natural Science Foundation of Hubei Province of China (2009CDA161). |
| Wardeh, Baylis, & Blagrove (2021) | M.W. acknowledges the support from BBSRC and MRC for the National Productivity Investment Fund (NPIF) fellowship (MR/R024898/1). M.W. and M.S.C.B acknowledge support from BBSRC IAA COVID - 168478. Establishment of the EID2 database was funded by a UK Research Council Grant (NE/G002827/1) to M.B., as part of an ERANET Environmental Health award to M.B.; subsequently, it has been further developed and maintained by BBSRC Tools and Resources Development Fund awards (BB/ K003798/1; BB/N02320X/1) to M.B., and the National Institute for Health Research Health Protection Research Unit (NIHR HPRU) in Emerging and Zoonotic Infections at the University of Liverpool in partnership with Public Health England and Liverpool School of Tropical Medicine. |
| Y. Xu & Wajtczak (2021) | This work is supported by the University of Liverpool. |
| B. Xu, Tan, Li, Jiang, & Peng (2017) | This study was supported by the National Key Plan for Scientific Research and Development of China (2016YFC1200204 and 2016YFD0500300), and the National Natural Science Foundation (31500126 and 31371338). |
| Yang et al. (2021) | We would like to acknowledge the financial support provided by the Natural Science Foundation of China (NSFC) under Grant No. 61876166, No.61663046 and No.91849132, CAMS Innovation Fund for Medical Sciences (2018-I2M-1-002), Beijing Hospital Nova Project (BJ-2018- 139), Yunnan provincial major science and technology special plan projects: digitization research and application demonstration of Yunnan characteristic industry, under Grant: 202002AD080001. |
| Yin, Zhou, Rashid, & Kwoh (2020) | This project is supported by AcRF Tier 2 grant MOE2014-T2-2-023, Ministry of Education, Singapore. |
| Young, Rogers & Robertson (2020) | FY is supported by a studentship from the Medical Research Council (MRC). DLR is funded by the MRC (MC_UU_1201412). |
| Zhang et al. (2019) | National Key Plan for Scientific Research and Development of China, Grant/Award Number: 2016YFD0500300; Hunan Provincial Natural Science Foundation of China, Grant/Award Number: 2018JJ3039; Changsha Science and Technology Bureau, Grant/Award Number: kq1801040; National Natural Science Foundation of China, Grant/ Award Number: 31500126 and 31671371; Chinese Academy of Medical Sciences, Grant/Award Number: 2016-I2M-1-005. |

*If no funding source was listed then listed as “No Funding Source Stated”. Otherwise, direct funding statements copied from primary text.

**References**

Aguas, R., & Ferguson, N. M. (2013). Feature Selection Methods for Identifying Genetic Determinants of Host Species in RNA Viruses. *PLoS Computational Biology*, *9*(10), e1003254. https://doi.org/10.1371/journal.pcbi.1003254

Ali, S., Bello, B., Chourasia, P., Punathil, R. T., Zhou, Y., & Patterson, M. (2022). PWM2Vec: An Efficient Embedding Approach for Viral Host Specification from Coronavirus Spike Sequences. *Biology*, *11*(3), 418. https://doi.org/10.3390/biology11030418

Attaluri, P. K., Chen, Z., & Lu, G. (2010). Applying neural networks to classify influenza virus antigenic types and hosts. *2010 IEEE Symposium on Computational Intelligence in Bioinformatics and Computational Biology*, 1–6. IEEE. https://doi.org/10.1109/CIBCB.2010.5510726

Attaluri, P. K., Zheng, X., Chen, Z., & Lu, G. (2009). *Applying machine learning techniques to classify H1N1 viral strains occurring in 2009 flu pandemic*. Retrieved from https://www.researchgate.net/publication/253864701_Applying_machine_learning_techniques_to_classify_H1N1_viral_strains_occurring_in_2009_flu_pandemic

Babayan, S. A., Orton, R. J., & Streicker, D. G. (2018). Predicting reservoir hosts and arthropod vectors from evolutionary signatures in RNA virus genomes. *Science*, *362*(6414), 577–580. https://doi.org/10.1126/science.aap9072

Bartoszewicz, J. M., Seidel, A., & Renard, B. Y. (2021). Interpretable detection of novel human viruses from genome sequencing data. *NAR Genomics and Bioinformatics*, *3*(1). https://doi.org/10.1093/nargab/lqab004

Bergner, L. M., Mollentze, N., Orton, R. J., Tello, C., Broos, A., Biek, R., & Streicker, D. G. (2021). Characterizing and Evaluating the Zoonotic Potential of Novel Viruses Discovered in Vampire Bats. *Viruses*, *13*(2), 252. https://doi.org/10.3390/v13020252

Brierley, L., & Fowler, A. (2021). Predicting the animal hosts of coronaviruses from compositional biases of spike protein and whole genome sequences through machine learning. *PLOS Pathogens*, *17*(4), e1009149. https://doi.org/10.1371/journal.ppat.1009149

Chrysostomou, C., Alexandrou, F., Nicolaou, M. A., & Seker, H. (2021). *Classification of influenza hemagglutinin protein sequences using convolutional neural networks*. Computation-based Science and Technology Research Center, The Cyprus Institute, Nicosia, CyprusThe Faculty of Computing, Engineering and the Built Environment, Birmingham City University, Birmingham; B5 5JU, United Kingdom: arXiv. https://doi.org/https://doi.org/10.48550/arXiv.2108.04240

Cook, P. W., Stark, T., Jones, J., Kondor, R., Zanders, N., Benfer, J., … Davis, C. T. (2020). Detection and Characterization of Swine Origin Influenza A(H1N1) Pandemic 2009 Viruses in Humans following Zoonotic Transmission. *Journal of Virology*, *95*(2). https://doi.org/10.1128/JVI.01066-20

Davis, P., & Russell, J. A. (2021). A Genotype-to-Phenotype Modeling Framework to Predict Human Pathogenicity of Novel Coronaviruses. *BioRxiv*, 2021.09.18.460926. https://doi.org/10.1101/2021.09.18.460926

Dhanoa, J., Manicassamy, B., & Chattopadhyay, I. (2018). Algorithmic Bio-surveillance For Precise Spatio-temporal Prediction of Zoonotic Emergence. *ArXiv*, 8 pp. https://doi.org/https://doi.org/10.48550/arXiv.1801.07807

ElHefnawi, M., & Sherif, F. F. (2014). Accurate classification and hemagglutinin amino acid signatures for influenza A virus host-origin association and subtyping. *Virology*, *449*, 328–338. https://doi.org/10.1016/j.virol.2013.11.010

Eng, C. L., Tong, J. C., & Tan, T. W. (2014). Predicting host tropism of influenza A virus proteins using random forest. *BMC Medical Genomics*, *7*(S3), S1. https://doi.org/10.1186/1755-8794-7-S3-S1

Eng, C. L., Tong, J. C., & Tan, T. W. (2016). Distinct host tropism protein signatures to identify possible zoonotic influenza a viruses. *PLoS ONE*, *11*(2), 1–12. https://doi.org/10.1371/journal.pone.0150173

Eng, C. L., Tong, J. C., & Tan, T. W. (2017). Predicting Zoonotic Risk of Influenza A Viruses from Host Tropism Protein Signature Using Random Forest. *International Journal of Molecular Sciences*, *18*(6), 1135. https://doi.org/10.3390/ijms18061135

Guo, Q., Li, M., Wang, C., Guo, J., Jiang, X., Tan, J., … Zhu, H. (2021). Predicting hosts based on early SARS-CoV-2 samples and analyzing the 2020 pandemic. *Scientific Reports*, *11*(1), 17422. https://doi.org/10.1038/s41598-021-96903-6

Guo, Q., Li, M., Wang, C., Wang, P., Fang, Z., tan, J., … Zhu, H. (2020). Host and infectivity prediction of Wuhan 2019 novel coronavirus using deep learning algorithm. *BioRxiv*, 2020.01.21.914044. https://doi.org/10.1101/2020.01.21.914044

Hu, W. (2010a). Host markers and correlated mutations in the overlapping genes of influenza viruses: M1, M2; NS1, NS2; and PB1, PB1-F2. *Natural Science*, *02*(11), 1225–1246. https://doi.org/10.4236/ns.2010.211150

Hu, W. (2010b). Novel host markers in the 2009 pandemic H1N1 influenza a virus. *Journal of Biomedical Science and Engineering*, *03*(06), 584–601. https://doi.org/10.4236/jbise.2010.36081

Hu, W. (2010c). Nucleotide host markers in the influenza A viruses. *Journal of Biomedical Science and Engineering*, *03*(07), 684–699. https://doi.org/10.4236/jbise.2010.37093

Kargarfard, F., Sami, A., Mohammadi-Dehcheshmeh, M., & Ebrahimie, E. (2016). Novel approach for identification of influenza virus host range and zoonotic transmissible sequences by determination of host-related associative positions in viral genome segments. *BMC Genomics*, *17*(1), 925. https://doi.org/10.1186/s12864-016-3250-9

Kincaid, C. (2018). N-Gram Methods for Influenza Host Classification. *Proceedings of the International Conference on Bioinformatics & Computational Biology (BIOCOMP)*, 105–107. Athens, Georgia, USA: The Steering Committee of The World Congress in Computer Science, Computer Engineering and Applied Computing (WorldComp). Retrieved from https://subzero.lib.uoguelph.ca/login?URL=?url=https://www.proquest.com/conference-papers-proceedings/n-gram-methods-influenza-host-classification/docview/2139460023/se-2?accountid=11233

King, D., Miller, Z., Jones, W., & Hu, W. (2010). Characteristic sites in the internal proteins of avian and human influenza viruses. *Journal of Biomedical Science and Engineering*, *03*(10), 943–955. https://doi.org/10.4236/jbise.2010.310125

Kou, Z., Lei, F., Wang, S., Zhou, Y., & Li, T. (2008). Molecular patterns of avian influenza A viruses. *Science Bulletin*, *53*(13), 2002–2007. https://doi.org/10.1007/s11434-008-0236-2

Kou, Z., Li, J., Fan, X., Kosari, S., & Qiang, X. (2021). Predicting Cross-Species Infection of Swine Influenza Virus with Representation Learning of Amino Acid Features. *Computational and Mathematical Methods in Medicine*, *2021*, 1–12. https://doi.org/10.1155/2021/6985008

Kuzmin, K., Adeniyi, A. E., DaSouza, A. K., Lim, D., Nguyen, H., Molina, N. R., … Harrison, R. W. (2020). Machine learning methods accurately predict host specificity of coronaviruses based on spike sequences alone. *Biochemical and Biophysical Research Communications*, *533*(3), 553–558. https://doi.org/10.1016/j.bbrc.2020.09.010

Kwon, E., Cho, M., Kim, H., & Son, H. S. (2020). A Study on Host Tropism Determinants of Influenza Virus Using Machine Learning. *Current Bioinformatics*, *15*(2), 121–134. https://doi.org/10.2174/1574893614666191104160927

Lee, B., Smith, D. K., & Guan, Y. (2021). Alignment free sequence comparison methods and reservoir host prediction. *Bioinformatics*, *37*(19), 3337–3342. https://doi.org/10.1093/bioinformatics/btab338

Li, H., & Sun, F. (2018). Comparative studies of alignment, alignment-free and SVM based approaches for predicting the hosts of viruses based on viral sequences. *Scientific Reports*, *8*(1), 10032. https://doi.org/10.1038/s41598-018-28308-x

Li, J., Zhang, S., Li, B., Hu, Y., Kang, X.-P., Wu, X.-Y., … Jiang, T. (2020). Machine Learning Methods for Predicting Human-Adaptive Influenza A Viruses Based on Viral Nucleotide Compositions. *Molecular Biology and Evolution*, *37*(4), 1224–1236. https://doi.org/10.1093/molbev/msz276

Meroz, D., Yoon, S. W., Ducatez, M. F., Fabrizio, T. P., Webby, R. J., Hertz, T., & Ben-Tal, N. (2011). Putative amino acid determinants of the emergence of the 2009 influenza A (H1N1) virus in the human population. *Proceedings of the National Academy of Sciences of the United States of America*, *108*(33), 13522–13527. https://doi.org/10.1073/PNAS.1014854108/-/DCSUPPLEMENTAL

Mock, F., Viehweger, A., Barth, E., & Marz, M. (2021). VIDHOP, viral host prediction with deep learning. *Bioinformatics (Oxford, England)*, *37*(3), 318–325. https://doi.org/10.1093/bioinformatics/btaa705

Mollentze, N., Babayan, S. A., & Streicker, D. G. (2021). Identifying and prioritizing potential human-infecting viruses from their genome sequences. *PLOS Biology*, *19*(9), e3001390. https://doi.org/10.1371/journal.pbio.3001390

Qiang, X., & Kou, Z. (2010). Prediction of interspecies transmission for avian influenza A virus based on a back-propagation neural network. *Mathematical and Computer Modelling*, *52*(11–12), 2060–2065. https://doi.org/10.1016/j.mcm.2010.06.008

Qiang, X., & Kou, Z. (2019). Scoring amino acid mutation to predict pandemic risk of avian influenza virus. *BMC Bioinformatics*, *20*(8), 288 (11 pp.). https://doi.org/10.1186/s12859-019-2770-0

Scarafoni, D., Telfer, B. A., Ricke, D. O., Thornton, J. R., & Comolli, J. (2019). Predicting Influenza A Tropism with End-to-End Learning of Deep Networks. *Health Security*, *17*(6), 468–476. https://doi.org/10.1089/hs.2019.0055

Shaltout, N. A., El-Hefnawi, M., Rafea, A., & Moustafa, A. (2014). Information Gain as a Feature Selection Method for the Efficient Classification of Influenza Based on Viral Hosts. *Proceedings of the World Congress on Engineering*. London. Retrieved from https://www.researchgate.net/publication/286743906_Information_Gain_as_a_Feature_Selection_Method_for_the_Efficient_Classification_of_Influenza_Based_on_Viral_Hosts

Shaltout, N., Rafea, A., Moustafa, A., & ElHefnawi, M. (2015). Using Information Gain to Compare the Effeciency of Machine Learning Techniques When Classifying Influenza Based on Viral Hosts. In *Transactions on Engineering Technologies* (pp. 707–722). Dordrecht: Springer Netherlands. https://doi.org/10.1007/978-94-017-9804-4_50

Sherif, F. F., Zayed, N., & Fakhr, M. (2017). Classification of host origin in influenza a virus by transferring protein sequences into numerical feature vectors. *International Journal of Biology and Biomedical Engineering*, *11*, 61–65. Retrieved from http://www.ncbi.nlm.nih.gov/genomes/

Sun, Y., Zhang, K., Qi, H., Zhang, H., Zhang, S., Bi, Y., … Li, J. (2021). Computational predicting the human infectivity of H7N9 influenza viruses isolated from avian hosts. *Transboundary and Emerging Diseases*, *68*(2), 846–856. https://doi.org/10.1111/tbed.13750

Sutanto, K., & Turcotte, M. (2021). Extracting and Evaluating Features from RNA Virus Sequences to Predict Host Species Susceptibility Using Deep Learning. *2021 13th International Conference on Bioinformatics and Biomedical Technology*, 81–89. New York, NY, USA: ACM. https://doi.org/10.1145/3473258.3473271

Tang, Q., Song, Y., Shi, M., Cheng, Y., Zhang, W., & Xia, X.-Q. (2015). Inferring the hosts of coronavirus using dual statistical models based on nucleotide composition. *Scientific Reports*, *5*(1), 17155. https://doi.org/10.1038/srep17155

Wang, J., Kou, Z., Duan, M., Ma, C., & Zhou, Y. (2013). Using Amino Acid Factor Scores to Predict Avian-to-Human Transmission of Avian Influenza Viruses: A Machine Learning Study. *Protein & Peptide Letters*, *20*(10), 1115–1121. https://doi.org/10.2174/0929866511320100005

Wang, J., Ma, C., Kou, Z., Zhou, Y. H., & Liu, H. L. (2013). Predicting transmission of avian influenza A viruses from avian to human by using informative physicochemical properties. *International Journal of Data Mining and Bioinformatics*, *7*(2), 166. https://doi.org/10.1504/IJDMB.2013.053198

Wardeh, M., Baylis, M., & Blagrove, M. S. C. (2021). Predicting mammalian hosts in which novel coronaviruses can be generated. *Nature Communications*, *12*(1), 780. https://doi.org/10.1038/s41467-021-21034-5

Xu, B., Tan, Z., Li, K., Jiang, T., & Peng, Y. (2017). Predicting the host of influenza viruses based on the word vector. *PeerJ*, *5*(7), e3579. https://doi.org/10.7717/peerj.3579

Xu, Y., & Wojtczak, D. (2021). Predicting Influenza A Viral Host Using PSSM and Word Embeddings. *2021 IEEE Conference on Computational Intelligence in Bioinformatics and Computational Biology (CIBCB)*, 1–10. University of Liverpool, Department of Computer Science, United Kingdom BT  - 2021 IEEE Conference on Computational Intelligence in Bioinformatics and Computational Biology (CIBCB), 13-15 Oct. 2021: IEEE. https://doi.org/10.1109/CIBCB49929.2021.9562959

Yang, Y., Guo, J., Wang, P., Wang, Y., Yu, M., Wang, X., … Sun, L. (2021). Reservoir hosts prediction for COVID-19 by hybrid transfer learning model. *Journal of Biomedical Informatics*, *117*, 103736. https://doi.org/10.1016/j.jbi.2021.103736

Yerukala Sathipati, S., Shukla, S. K., & Ho, S.-Y. (2022). Tracking the amino acid changes of spike proteins across diverse host species of severe acute respiratory syndrome coronavirus 2. *IScience*, *25*(1), 103560. https://doi.org/10.1016/j.isci.2021.103560

Yin, R., Zhou, X., Rashid, S., & Kwoh, C. K. (2020). HopPER: an adaptive model for probability estimation of influenza reassortment through host prediction. *BMC Medical Genomics*, *13*(1), 9. https://doi.org/10.1186/s12920-019-0656-7

Young, F., Rogers, S., & Robertson, D. L. (2020). Predicting host taxonomic information from viral genomes: A comparison of feature representations. *PLOS Computational Biology*, *16*(5), e1007894. https://doi.org/10.1371/journal.pcbi.1007894

Zhang, Z., Cai, Z., Tan, Z., Lu, C., Jiang, T., Zhang, G., & Peng, Y. (2019). Rapid identification of human‐infecting viruses. *Transboundary and Emerging Diseases*, *66*(6), 2517–2522. https://doi.org/10.1111/tbed.13314
