# Supplementary material for: Predicting host species susceptibility to influenza viruses and coronaviruses using genome data and machine learning: a scoping review
Source: Front Vet Sci. 2024 Sep 25;11:1358028. doi: 10.3389/fvets.2024.1358028 (PMC11462629; doi:10.3389/fvets.2024.1358028)
Supplement: Supplementary file 4 [file Table_4.DOCX]

Table S4: Author Department Categorizations Used

| Category | Departments Included |
| --- | --- |
|  |  |
| Agriculture | Agriculture |
|  |  |
| Biological Sciences | Biochemistry  Biodiversity  Biological Science  Molecular and Cellular Biology  Systems Biology |
|  |  |
| Computer Science | Computer Science  Software |
|  |  |
| Data Science | Bioinformatics  Health Data Science  Informatics and Systems Department and Biomedical Informatics and Cheminformatics group |
|  |  |
| Engineering | Biomedical Engineering  Digital Engineering |
|  |  |
| Epidemiology | Epidemiology |
|  |  |
| Health | Animal Health and Comparative Medicine  Public Health  Livestock and One Health  Medicine |
|  |  |
| Mathematics and Statistics | Mathematics and Statistics |
|  |  |
| Microbiology | Virology  Microbiology and Immunology |
|  |  |
| Not Stated | Not Stated |
|  |  |
