# Supplementary material for: Predicting host species susceptibility to influenza viruses and coronaviruses using genome data and machine learning: a scoping review
Source: Front Vet Sci. 2024 Sep 25;11:1358028. doi: 10.3389/fvets.2024.1358028 (PMC11462629; doi:10.3389/fvets.2024.1358028)
Supplement: Supplementary file 5 [file Table_5.DOCX]

Table S5: Publications in which viruses other than influenza or coronaviruses were used by authors.

| Reference | Viruses used* |
| --- | --- |
| Aguas & Ferguson (2013) | Flavivirus and Alphavirus geneses, and Calciviridae and Paramyxoviridae families and rabies virus. |
|  |  |
| Babayan et al. (2018) | Togavirus, Rhabdovirus, Picornavirus, Paramyxovirus, Hepevirus, Flavivirus, Filovirus, Calicivirus, Bunyavirus, Astrovirus, Arenavirus, and Zaire Ebolavirus epidemic dataset. |
|  |  |
| Bartoszewicz et al.(2021) | We accessed the Virus-Host Database on July 31, 2019, and downloaded all the available data. |
| Bergner et al. (2021) | One known zoonosis, rabies virus (RABV, Rabies lyssavirus, family Rhabdoviridae), and putatively novel representatives from the families Hepeviridae, Coronaviridae, Reoviridae, Astroviridae and Picornaviridae with unknown human infectivity. |
|  |  |
| Guo et al. (2021) | Not Stated |
|  |  |
| Lee, Smith, and Guan (2021) | 437 viral species [as developed in (Babayan, et al., 2018) were included]. |
| Li & Sun (2018b) | Rabies |
|  |  |
| Mock et al. (2021) | Rotavirus A, Rabies |
|  |  |
| Sutanto & Turcotte (2021) | Our dataset contains sequences of RNA viruses and their host species. It was obtained from NCBI Virus on September 12, 2020. |
| Xu et al. (2017) | Swissprot dataset – unclear what viruses this includes. |
| Yang et al. (2021) | Arenavirus, Astrovirus, Bunyavirus, Calicivirus, Coronavirus, Filovirus, Flavivirus, Hepevirus, Paramyxovirus, Picornavirus, Rhabdovirus |
| Young, Rogers, and Robertson (2020) | We downloaded the Virus Host Database (https://www.genome.jp/virushostdb/) on 25/1/ 2019. 9199 viruses were included. |
|  |  |
| Zhang (2019) | Not Stated |

*As reported by authors.

References

Aguas, Ricardo, and Neil M. Ferguson. 2013. “Feature Selection Methods for Identifying Genetic Determinants of Host Species in RNA Viruses.” Edited by Sergei L. Kosakovsky Pond. *PLoS Computational Biology* 9 (10): e1003254. https://doi.org/10.1371/journal.pcbi.1003254.

Babayan, Simon A., Richard J. Orton, and Daniel G. Streicker. 2018. “Predicting Reservoir Hosts and Arthropod Vectors from Evolutionary Signatures in RNA Virus Genomes.” *Science* 362 (6414): 577–80. https://doi.org/10.1126/science.aap9072.

Bartoszewicz, Jakub M, Anja Seidel, and Bernhard Y Renard. 2021. “Interpretable Detection of Novel Human Viruses from Genome Sequencing Data.” *NAR Genomics and Bioinformatics* 3 (1). https://doi.org/10.1093/nargab/lqab004.

Bergner, Laura M., Nardus Mollentze, Richard J. Orton, Carlos Tello, Alice Broos, Roman Biek, and Daniel G. Streicker. 2021. “Characterizing and Evaluating the Zoonotic Potential of Novel Viruses Discovered in Vampire Bats.” *Viruses* 13 (2): 252. https://doi.org/10.3390/v13020252.

Guo, Qian, Mo Li, Chunhui Wang, Jinyuan Guo, Xiaoqing Jiang, Jie Tan, Shufang Wu, et al. 2021. “Predicting Hosts Based on Early SARS-CoV-2 Samples and Analyzing the 2020 Pandemic.” *Scientific Reports* 11 (1): 17422. https://doi.org/10.1038/s41598-021-96903-6.

Lee, Bill, David K Smith, and Yi Guan. 2021. “Alignment Free Sequence Comparison Methods and Reservoir Host Prediction.” Edited by Jonathan Wren. *Bioinformatics* 37 (19): 3337–42. https://doi.org/10.1093/bioinformatics/btab338.

Li, Han, and Fengzhu Sun. 2018. “Comparative Studies of Alignment, Alignment-Free and SVM Based Approaches for Predicting the Hosts of Viruses Based on Viral Sequences.” *Scientific Reports* 8 (1): 10032. https://doi.org/10.1038/s41598-018-28308-x.

Mock, Florian, Adrian Viehweger, Emanuel Barth, and Manja Marz. 2021. “VIDHOP, Viral Host Prediction with Deep Learning.” *Bioinformatics (Oxford, England)* 37 (3): 318–25. https://doi.org/10.1093/bioinformatics/btaa705.

Sutanto, Kevin, and Marcel Turcotte. 2021. “Extracting and Evaluating Features from RNA Virus Sequences to Predict Host Species Susceptibility Using Deep Learning.” In *2021 13th International Conference on Bioinformatics and Biomedical Technology*, 81–89. ACM International Conference Proceeding Series. New York, NY, USA: ACM. https://doi.org/10.1145/3473258.3473271.

Xu, Beibei, Zhiying Tan, Kenli Li, Taijiao Jiang, and Yousong Peng. 2017. “Predicting the Host of Influenza Viruses Based on the Word Vector.” *PeerJ* 5 (7): e3579. https://doi.org/10.7717/peerj.3579.

Yang, Yun, Jing Guo, Pei Wang, Yaowei Wang, Minghao Yu, Xiang Wang, Po Yang, and Liang Sun. 2021. “Reservoir Hosts Prediction for COVID-19 by Hybrid Transfer Learning Model.” *Journal of Biomedical Informatics* 117 (May):103736. https://doi.org/10.1016/j.jbi.2021.103736.

Young, Francesca, Simon Rogers, and David L. Robertson. 2020. “Predicting Host Taxonomic Information from Viral Genomes: A Comparison of Feature Representations.” Edited by Morgan Langille. *PLOS Computational Biology* 16 (5): e1007894. https://doi.org/10.1371/journal.pcbi.1007894.

Zhang, Zheng, Zena Cai, Zhiying Tan, Congyu Lu, Taijiao Jiang, Gaihua Zhang, and Yousong Peng. 2019. “Rapid Identification of Human‐infecting Viruses.” *Transboundary and Emerging Diseases* 66 (6): 2517–22. https://doi.org/10.1111/tbed.13314.
