# Supplementary material for: Predicting host species susceptibility to influenza viruses and coronaviruses using genome data and machine learning: a scoping review
Source: Front Vet Sci. 2024 Sep 25;11:1358028. doi: 10.3389/fvets.2024.1358028 (PMC11462629; doi:10.3389/fvets.2024.1358028)
Supplement: Supplementary file 6 [file Table_6.DOCX]

Table S6. Species used in analyses.*

| Species^†^ | Number of Analysis (All Viruses)  (n = 77) | | Number of Analysis (Corona-viruses)  (n = 23) | Number of Analysis (Influenza Viruses)  (n = 42) | | Number of Analysis (Other Viruses)  (n =19) | |
| --- | --- | --- | --- | --- | --- | --- | --- |
|  |  |  | | |  | |  |
| Human | 57 | 15 | | | 36 | | 6 |
| Avian | 35 | 4 | | | 31 | | 0 |
| Swine | 28 | 3 | | | 25 | | 0 |
| Non-Human | 9 | 5 | | | 1 | | 3 |
| Pandemic Human | 3 | 0 | | | 3 | | 0 |
| Zoonotic | 3 | 0 | | | 3 | | 0 |
| Bovine | 2 | 2 | | | 0 | | 0 |
| *E. fuscus* | 2 | 0 | | | 0 | | 2 |
| Environment | 2 | 1 | | | 1 | | 0 |
| Equine | 2 | 1 | | | 1 | | 0 |
| *L. borealis* | 2 | 0 | | | 0 | | 2 |
| *L. cinereus* | 2 | 0 | | | 0 | | 2 |
| *L. seminolus* | 2 | 0 | | | 0 | | 2 |
| Mammals | 2 | 2 | | | 0 | | 0 |
| *T. brasiliensis* | 2 | 0 | | | 0 | | 2 |
| All non-human (including bacteria) | 1 | 0 | | | 0 | | 1 |
| All non-human Chordata | 1 | 0 | | | 0 | | 1 |
| *Anas acuta* | 1 | 0 | | | 1 | | 0 |
| *Anas carolinensis* | 1 | 0 | | | 1 | | 0 |
| *Anas clypeata* | 1 | 0 | | | 1 | | 0 |
| *Anas discors* | 1 | 0 | | | 1 | | 0 |
| *Anas rubripes* | 1 | 0 | | | 1 | | 0 |
| *Antrozous Pallidus* | 1 | 0 | | | 0 | | 1 |
| *Arenaria interpres* | 1 | 0 | | | 1 | | 0 |
| Bat | 1 | 1 | | | 0 | | 0 |
| Camel | 1 | 1 | | | 0 | | 0 |
| Canine | 1 | 0 | | | 1 | | 0 |
| Cat | 1 | 1 | | | 0 | | 0 |
| *Corynorhinus townsendii* | 1 | 0 | | | 0 | | 1 |
| Dog | 1 | 1 | | | 0 | | 0 |
| Dolphin | 1 | 1 | | | 0 | | 0 |
| Feline | 1 | 0 | | | 1 | | 0 |
| Fish | 1 | 1 | | | 0 | | 0 |
| Hedgehog | 1 | 1 | | | 0 | | 0 |
| *Lasionycteris Noctivagans* | 1 | 0 | | | 0 | | 1 |
| *Lasiurus Blossevilii* | 1 | 0 | | | 0 | | 1 |
| *Lasiurus intermedius* | 1 | 0 | | | 0 | | 1 |
| *Lasiurus xanthinus* | 1 | 0 | | | 0 | | 1 |
| Monkey | 1 | 1 | | | 0 | | 0 |
| Murine | 1 | 1 | | | 0 | | 0 |
| *Myotis Austroriparius* | 1 | 0 | | | 0 | | 1 |
| *Myotis californicus* | 1 | 0 | | | 0 | | 1 |
| *Myotis evotis* | 1 | 0 | | | 0 | | 1 |
| *Myotis lucifugus* | 1 | 0 | | | 0 | | 1 |
| *Myotis yumanensis* | 1 | 0 | | | 0 | | 1 |
| Non-mammals | 1 | 1 | | | 0 | | 0 |
| Not Stated | 1 | 0 | | | 0 | | 1 |
| *Nycticeius humeralis* | 1 | 0 | | | 0 | | 1 |
| Palm civet | 1 | 1 | | | 0 | | 0 |
| Pangolin | 1 | 1 | | | 0 | | 0 |
| *Parastrellus hesperus* | 1 | 0 | | | 0 | | 1 |
| *Perimyotis subflavus* | 1 | 0 | | | 0 | | 1 |
| Porcine | 1 | 1 | | | 0 | | 0 |
| Python | 1 | 1 | | | 0 | | 0 |
| Rat | 1 | 1 | | | 0 | | 0 |
| Turtle | 1 | 1 | | | 0 | | 0 |
| Weasel | 1 | 1 | | | 0 | | 0 |

* Analyses can use a combination of multiple taxonomic levels.
† The definition for species level was non-strict since some publications defined species to a non-species level (i.e., non-human, avian, bovine, etc.). Species were recorded as reported by authors.
