# Supplementary material for: Predicting host species susceptibility to influenza viruses and coronaviruses using genome data and machine learning: a scoping review
Source: Front Vet Sci. 2024 Sep 25;11:1358028. doi: 10.3389/fvets.2024.1358028 (PMC11462629; doi:10.3389/fvets.2024.1358028)
Supplement: Supplementary file 7 [file Table_7.DOCX]

Table S7. Host Specie’s taxonomic levels used in analyses.

| **Taxonomic Level** | **Number of analyses*** |
| --- | --- |
|  |  |
| Species | 69 |
| Order | 14 |
| Class | 11 |
| Kingdom | 9 |
| Family | 8 |
| Phylum | 5 |
| Domain | 4 |
| Suborder | 4 |
| Genus | 3 |
| Not Stated | 2 |

* Analyses can use a combination of multiple taxonomic levels.
