# Supplementary material for: Predicting host species susceptibility to influenza viruses and coronaviruses using genome data and machine learning: a scoping review
Source: Front Vet Sci. 2024 Sep 25;11:1358028. doi: 10.3389/fvets.2024.1358028 (PMC11462629; doi:10.3389/fvets.2024.1358028)
Supplement: Supplementary file 8 [file Table_8.DOCX]

Table S8: Categories of Sequence Formats

| Category | Sequence Formats Included | Classification* |
| --- | --- | --- |
|  |  |  |
| Genomic Bias | Dinucleotide Bias | N |
|  | Genomic Bias | N |
|  | Codon Pair Bias | A |
|  | Codon Usage Bias | A |
|  | Genome Composition-Based | N |
|  |  |  |
| Encoding Scheme | Hot Encoding | B |
|  | Direct Encoding Scheme | N |
|  | Numeric Sequence | N |
|  |  |  |
| Sequence Length | Normal Repeat With Gaps | N |
|  | Random Repeat | N |
|  | Random Repeat With Gaps | N |
|  | Smallest - All Cut To Shortest Length | N |
|  | Short Reads | N |
|  | Append Gaps | N |
|  | Receptor | N |
|  |  |  |
| Frequency | AA Composition | A |
|  | Mononucleotide Frequency | N |
|  | Dipeptide Composition | A |
|  | PSSM Eg - Position Weight | A |
|  | PSSM Er –Position Weight | A |
|  | AA Factor | A |
|  | PWM2VEC Feature Vector | A |
|  |  |  |
| Substring Frequency | K-Mer | B |
|  | Skip-Mer | B |
|  | N-Gram | A |
|  | Word Embedding | A |
|  | Word Vector | A |
|  |  |  |
| Pseudo Composition | Parallel Correlation-Based Pseudo-Amino-Acid Composition | A |
|  | Pseudo AA Composition | A |
|  | Position Entropy | A |
|  | Node Energy Coefficients | A |
|  |  |  |
| Physicochemical Properties | Amino Acid Physicochemical Properties | A |
|  | AA Hydrophobicity Index | A |
|  | Host Tropism Protein Signature Feature Transformation | A |
|  |  |  |
| Structural Pattern | M-Sp | B |
|  | Secondary Structures | B |
|  | Secondary Structure Fingerprints | B |
|  | Domains | B |
|  |  |  |
| Other | Phylogenetic Neighbourhood | B |
|  | Similarity To Human Genome Transcripts | B |
|  | Truncated Singular Value Decomposition (Tsvd) - As Dimensionality Reduction | A |
|  | Online – Online Deep Learning To Select More Sequences | A |
|  |  |  |
| Unchanged | Nucleotide Sequence | N |
|  | Amino Acid Sequence | A |
|  |  |  |
| Not Stated | Not Stated |  |
|  |  |  |

*Classification was determined by the most common sequence format used. The format used in the corresponding figure was determined based on whether Nucleotide Sequence (N) or Amino Acid Sequence (A) or Both (B) were selected in the corresponding input sequence question if no selection was made the classification defaulted to that used in this table.
