# Supplementary material for: Predicting host species susceptibility to influenza viruses and coronaviruses using genome data and machine learning: a scoping review
Source: Front Vet Sci. 2024 Sep 25;11:1358028. doi: 10.3389/fvets.2024.1358028 (PMC11462629; doi:10.3389/fvets.2024.1358028)
Supplement: Supplementary file 9 [file Table_9.DOCX]

Table S9: Feature Selection Methods Used

| Feature Selection Classifier |  | Number of Classifiers  (n = 174)* | Percentage (%)* |
| --- | --- | --- | --- |
|  |  |  |  |
| Gradient Boosted Classification Tree |  | 15 | 8.6 |
| Random Forest As Feature Selection |  | 14 | 8.0 |
| Information Gain |  | 11 | 6.3 |
| Single Value Decomposition |  | 8 | 4.6 |
| Ridge Regression |  | 7 | 4.0 |
| Principal Component Analysis As Feature Selection |  | 5 | 2.9 |
| 1R |  | 4 | 2.3 |
| Chi-Squared Feature Selection |  | 4 | 2.3 |
| Filtered Attribute Evaluation |  | 4 | 2.3 |
| Information Entropy |  | 4 | 2.3 |
| Information Gain Ratio |  | 4 | 2.3 |
| Relief |  | 4 | 2.3 |
| Support Vector Machine As Feature Selection |  | 4 | 2.3 |
| Symmetric Uncertainty |  | 4 | 2.3 |
| Truncated Singular Value Decomposition |  | 4 | 2.3 |
| Correlation Feature Selection Classifier |  | 3 | 1.7 |
| Minimum Redundancy Maximum Relevance |  | 3 | 1.7 |
| Classification Tree As Feature Selection |  | 2 | 1.1 |
| Genetic Classifier |  | 2 | 1.1 |
| Machine-Learning Predicted Probability |  | 2 | 1.1 |
| Confidence-Based Autonomy As Feature Selection |  | 1 | 0.6 |
| Fusion Of Network Similarities |  | 1 | 0.6 |
| Inheritable Bi-Objective Combinatorial Genetic Classifier |  | 1 | 0.6 |
| Machine Learning With Digital Signal Processing |  | 1 | 0.6 |
| RIPPER As Feature Selection |  | 1 | 0.6 |
| Two Sample Logo |  | 1 | 0.6 |
| Variant Effect Score Prediction Without Alignments |  | 1 | 0.6 |
| Vorpal |  | 1 | 0.6 |
| Not Stated |  | 92 | 52.9 |

*Some classifiers may have multiple feature selection methods. (i.e., the sum is greater than 174 or 100%)
