# Supplementary material for: Predicting host species susceptibility to influenza viruses and coronaviruses using genome data and machine learning: a scoping review
Source: Front Vet Sci. 2024 Sep 25;11:1358028. doi: 10.3389/fvets.2024.1358028 (PMC11462629; doi:10.3389/fvets.2024.1358028)
Supplement: Supplementary file 10 [file Table_10.DOCX]

| Machine Learning Algorithm |  | Number of Classifiers (n=174)* | Percentage (%)* | Categorization** |
| --- | --- | --- | --- | --- |
|  |  |  |  |  |
| Random Forest |  | 31 | 17.8 | Random Forest |
| Support Vector Machine |  | 28 | 16.1 | Support Vector Machine |
| k-Nearest Neighbour |  | 15 | 8.6 | k-Nearest Neighbour |
| Classification Tree |  | 12 | 6.9 | Classification Tree |
| Naïve Bayes |  | 12 | 6.9 | Naïve Bayes |
| Gradient Boosting Machine |  | 9 | 5.2 | Gradient Boosting Machine |
| Logistic Regression |  | 9 | 5.2 | Logistic Regression |
| Bi-Path Convolutional Neural Networks |  | 8 | 4.6 | Neural Network |
| Long Short-Term Memory |  | 8 | 4.6 | Neural Network |
| Convolution Neural Network |  | 7 | 4.0 | Neural Network |
| Neural Network |  | 7 | 4.0 | Neural Network |
| Multilayer Perceptron |  | 5 | 2.9 | Neural Network |
| Part Of An ENSEMBLE |  | 5 | 2.9 | ENSEMBLE |
| Adaboost |  | 3 | 1.7 | Adaboost |
| Deep Neural Network |  | 3 | 1.7 | Neural Network |
| Extreme Gradient Boosting |  | 3 | 1.7 | Gradient Boosting |
| Rotation Forest |  | 3 | 1.7 | Random Forest |
| Alternating Classification Trees |  | 2 | 1.1 | Classification Tree |
| Hierarchal Clustering |  | 2 | 1.1 | Hierarchal Clustering |
| C4.5 |  | 1 | 0.6 | Classification Tree |
| Confidence-Based Autonomy |  | 1 | 0.6 | CBA |
| Class Weight-Biased Logistic Regression |  | 1 | 0.6 | Logistic Regression |
| Co-Training For Domain Adaptation |  | 1 | 0.6 | Co-Training |
| Deep Learning |  | 1 | 0.6 | Neural Network |
| Mahalanobis Distance Discriminant |  | 1 | 0.6 | Mahalanobis |
| Metric Transfer Learning |  | 1 | 0.6 | Transfer Learning |
| RIPPER |  | 1 | 0.6 | RIPPER |
| Rusboost |  | 1 | 0.6 | Rusboost |
| Sequential Minimal Optimization |  | 1 | 0.6 | Minimal Optimization |
| Simple Logistic |  | 1 | 0.6 | Logistic Regression |
| Stochastic Gradient Descent |  | 1 | 0.6 | Gradient Descent |
| Transfer Learning |  | 1 | 0.6 | Transfer Learning |

Table S10: Machine Learning Algorithm Used

*Some classifiers may have multiple algorithms. (i.e., the sum is greater than 174 or 100%)

** Categorizations used in Table 5
