# Supplementary material for: Predicting host species susceptibility to influenza viruses and coronaviruses using genome data and machine learning: a scoping review
Source: Front Vet Sci. 2024 Sep 25;11:1358028. doi: 10.3389/fvets.2024.1358028 (PMC11462629; doi:10.3389/fvets.2024.1358028)
Supplement: Supplementary file 11 [file Table_11.DOCX]

Table S11: Validation Techniques Used

| Validation Technique |  | Number of Classifiers  (n = 174)* | Percentage  (%) |
| --- | --- | --- | --- |
|  |  |  |  |
| N-fold cross-validation |  | 137 | 78.7 |
| Split into training and validation dataset |  | 50 | 28.7 |
| Split into training, optimization, and validation dataset |  | 34 | 19.5 |
| Simulation |  | 10 | 5.8 |
| Experimental validation |  | 9 | 5.2 |
| Independent dataset |  | 9 | 5.2 |
| Out-of-bag Validation |  | 6 | 3.4 |
| Comparison to previous results |  | 5 | 2.9 |
| Bootstrapping |  | 1 | 0.6 |
| Not stated |  | 11 | 6.3 |

*Some classifiers used multiple validation techniques (i.e., the sum is greater than 174 and 100%)
