# Supplementary material for: Predicting host species susceptibility to influenza viruses and coronaviruses using genome data and machine learning: a scoping review
Source: Front Vet Sci. 2024 Sep 25;11:1358028. doi: 10.3389/fvets.2024.1358028 (PMC11462629; doi:10.3389/fvets.2024.1358028)
Supplement: Supplementary file 12 [file Table_12.DOCX]

Table S12: Measures of accuracy used for analyses.

| Accuracy Measure |  | Number of Analysis (n=77)* | Percentage (%) |
| --- | --- | --- | --- |
|  |  |  |  |
| Raw accuracy |  | 55 | 75.3 |
| Sensitivity |  | 31 | 40.2 |
| Specificity |  | 28 | 36.4 |
| ROC** |  | 22 | 28.6 |
| AUC |  | 18 | 23.4 |
| F- Score*** |  | 14 | 18.2 |
| Positive Predictive Value |  | 13 | 16.9 |
| Feature Importance |  | 10 | 13.0 |
| Matthews Correlation Coefficient |  | 9 | 11.7 |
| Time |  | 4 | 5.2 |
| Balanced Accuracy |  | 3 | 3.9 |
| Out of Bag Error Rate |  | 3 | 3.9 |
| Area Under Precision-Recall Curve |  | 2 | 2.6 |
| Cohen’s Kappa Statistic |  | 2 | 2.6 |
| Confidence- In-class association |  | 1 | 1.3 |
| G-Means |  | 1 | 1.3 |
| Mean Squared Error |  | 1 | 1.3 |
| P-Value Cluster |  | 1 | 1.3 |
| Support Statistics |  | 1 | 1.3 |
| True Skill Statistic |  | 1 | 1.3 |
| Not Stated |  | 3 | 3.9 |

* Some analyses used multiple accuracy measures (i.e., the sum is greater than 174 and 100%)
** Includes weighted-ROC (n = 2)
*** Includes macro F-Score (n = 3) and Weighted F-Score (n = 1)
