# Supplementary material for: Predicting host species susceptibility to influenza viruses and coronaviruses using genome data and machine learning: a scoping review
Source: Front Vet Sci. 2024 Sep 25;11:1358028. doi: 10.3389/fvets.2024.1358028 (PMC11462629; doi:10.3389/fvets.2024.1358028)
Supplement: Supplementary file 15 [file Data_Sheet_2.pdf]

The Use of Machine Learning and Predictive Modelling Methods in the Identification of Hosts for Viral Infections: Scoping Review Protocol

Authors: Famke Alberts[1], Sheila Keay[1], Zvonimir Poljak[1]

Author Affiliations: [1]Department of Population Medicine, Ontario Veterinary College, University of Guelph, Guelph, Canada.

Abstract

Background: Advanced *in-silico* predictive modelling techniques combining methods of machine learning and bioinformatics have been applied to predict the reservoir of a virus and all hosts that exist within that reservoir. However, a systematic compilation of this body of research does not exist.

Objectives: This protocol describes the methods that will be used to conduct a formal scoping review of current literature to address the question: “What machine learning methods have been applied to influenza virus and coronavirus genome data for identification of the potential reservoirs?”.

Eligibility Criteria: Eligible studies will be primary research studies, in English, from any geographic location, published between 2000-2021, conducted using machine learning techniques within the context of understanding or predicting influenza virus or coronavirus host-range or transmission.

Sources of Evidence: The following databases will be searched: PubMed, MEDLINE, ProQuest, Engineering Village, and Web of Science from 2000-2021.

Charting Methods: We will extract data on general and specific study characteristics, identifying the steps taken in data gathering, processing, and analysis.

1. Introduction

1.1 Rationale

The transmission of pathogens between species is an important agricultural and public health concern with negative economic and health-related consequences. Practical disease control requires an understanding of pathogen reservoirs (Haydon et al., 2002). How a reservoir is defined may limit the inclusion of 'incidental hosts' (Ashford, 2003). Haydon's definition of a reservoir as a system accounts for this where, for example, a multi-host pathogen may be maintained within a community of both maintenance and non-maintenance populations, and is useful when integrating multiple data sources to understand reservoir dynamics and the impacts of control interventions (Viana et al., 2014). Haydon's definition of reservoir will be used, and the species contained within the reservoir, both target, and non-target species, will be defined as the host range.

Traditionally, virus hosts have been identified through traditional empirical evidence derivation using methods such as lab testing, surveillance, and other epidemiological evidence, including also phylogenetic analysis. While this work provides the essential basis for understanding factors of viral host range, application of newer strategies combining bioinformatics techniques with machine learning techniques are being applied to expand predictive capabilities for the emergence of viruses in new host species. Accuracy of prediction of these interspecies spill-over, or host-shift events, has implications for preparedness and responses to outbreaks (Dolan et al., 2018; Lee et al., 2021). For this review, influenza viruses and coronaviruses will be the focus because they are well-documented single-stranded RNA virus species that infect and have become established in multiple host species (Grange et al. 2021) All genus of influenza viruses (*Alphainfluenzavirus, Betainfluenzavirus, Gammainfluenzavirus,* and *Deltainfluenzavirus*) and coronaviruses (*Alphacoronavirus, Betacoronavirus, Gammacoronavirus,* and *Deltacoronavirus*) have been shown to exhibit multiple hosts.

Abd-Alrazaq (Abd-Alrazaq et al., 2020) published findings of a scoping review of uses of artificial intelligence (AI) technology during the recent COVID-19 pandemic and identified 3 publications using AI to predict potential hosts or reservoirs of SARS-CoV-2. Here we extend the scope of this review to include all genus of influenza viruses and coronaviruses. Additionally, the review will be expanded to include publications since the year 2000.

A scoping review of this topic could be useful for identifying the depth and breadth of this research and may be of use to those interested in pursuing such research, or to potential funding agencies and policymakers specialized in areas of either agricultural or public health preventative risk management (Geoghegan and Holmes, 2017).

1.2 Objectives

The objective of this protocol is to describe the methods that will be used to conduct a scoping review to address the following research question: "What machine learning methods have been applied to influenza virus and coronavirus genome data for identification of the potential reservoirs?". The scoping review will follow the methodological framework outlined by Arksey and O'Malley (2005).

2. **Methods**

2.1 Protocol and Registration

This protocol was prepared using the Preferred Reporting Items for Systematic Reviews and Meta-Analyses Extension for Scoping Reviews (PRISMA-ScR) reporting guidelines (Tricco et al. 2018) and will be published on the University of Guelph Atrium (https://atrium.lib.uoguelph.ca/).

2.2 Eligibility Criteria

To be eligible for inclusion in this review studies must be full-text English language publications of primary research from any geographic location published during or after 2000. The approaches outlined in the objectives of this review were not relevant prior to the introduction of whole genome sequencing and the advancement of machine learning techniques which, at the earliest, would have started occurring in 2000.

Population eligible/excluded:

Primary research investigating the host range of any virus given that the study is inclusive of coronaviruses or influenza viruses. Conference proceedings will be included due to the rapid evolvement of this area of research and the lag that may be present in the publication of peer-reviewed journal articles. All genus of coronavirus and influenza virus will be considered as each has been documented as capable of transmission in multiple host species; both are global public health and agricultural priority pathogens and have been extensively surveyed at the genomic level (Grange et al 2021).

Intervention eligible/excluded:

Research conducted using machine learning techniques on any genomic data for the purpose of understanding or predicting influenza virus or coronavirus host-range or transmission.

Anticipated protocol deviations:

It is anticipated that the literature available may be limited based on the scoping review performed by Abd-Alrazaq (Abd-Alrazaq et al., 2020) on the uses of artificial intelligence (AI) technology during the recent COVID-19 pandemic. In the case that the results are too limited the scope may be expanded to the scope of any relevant recent review publications that may be found during the completion of the initial search.

2.3 Information Sources

The following databases will be searched for relevant studies with a publication limit of 2000-2021:

Conference Proceedings/Abstracts:
The following conference proceedings/ abstracts will be hand-searched:

- International Conference on Computational Biology and Bioinformatics (ICCBB)- http://www.iccbb.org/.
    - Annual international conference. Titles and full-text conference papers are available for 2017-2020.
- Computational Intelligence Methods for Bioinformatics and Biostatistics (CIBB)- http://www.isa.cnr.it/cibb2021/
    - Annual international conference. Selected conference papers are available for 2008-2019.
- Intelligent Systems for Molecular Biology (ISMB)- https://www.iscb.org/about-ismb
    - Annual international conference. Titles and full proceedings are available for 1993-2020.

Bibliographic Database Search Strategy and vendor interfaces (platforms):

**Platform (vendor interface)**	**Database**
Elsevier	Engineering Village – Inspec and Compendex

NCBI (National Center for Biotechnology Information) website	PubMed – includes MEDLINE (National Library of Medicine biomedical database of citations and abstracts indexed using MeSH thesaurus))<br>- also includes in-process and other non-indexed citations (books, manuscripts, and articles supplied ahead of print)
ProQuest	Coronavirus Research Database
Web of Science (The Science Citation Index, Clarivate Analytics, 1864-current)	(multiple)
Ovid Technologies Inc.	MEDLINE (National Library of Medicine biomedical database of citations and abstracts indexed using MeSH thesaurus))

The selected results will be hand-searched for additional results within the references. The bibliographies of recent review publications on this topic and a selection of relevant manuscripts will be hand-searched for additional reports missed by our database search. All citations will be downloaded (or if necessary, manually added) to Mendeley reference management software (© 2021 Mendeley Ltd.) for deduplication. De-duplicated citations will be downloaded to Systematic Review and Literature Review software- Distiller-SR software package (Copyright © 2008-2021, Evidence Partners Inc.) for relevance screening and data characterization.

2.4 Search

Search strings will be developed and formatted for selected bibliometric platforms with support from a University of Guelph librarian with expertise and experience in scoping review methods. Search string keywords were developed and categorized into the three main categories involved in answering the main research question. These included the viral pathogens, the machine learning approach, and the host range of the pathogen.

<u>Example search string formulated in Ovid- MEDLINE:</u>

#	Query	Results from 26 Jul 2021

1	Influenza, Human/	52,352
2	exp Orthomyxoviridae/	59,751
3	exp Coronaviridae/	85,254
4	exp COVID-19/	92,178
5	exp Coronaviridae Infections/	103,666
6	(influenza* or Orthomyxovir* or flu or Coronavir* or covid or IAV).tw.	259,939
7	1 or 2 or 3 or 4 or 5 or 6	286,331
8	Host Specificity/	4,039
9	zoonoses/ or viral zoonoses/	17,836
10	Viral Tropism/	1,731
11	exp disease transmission, infectious/ or disease reservoirs/ or disease vectors/	87,533
12	(zoono* or between-species transmission or host range or cross-species transmission or pathogen spillover or spillover or host tropism or host specificity or reservoir).tw.	94,143
13	8 or 9 or 10 or 11 or 12	184,933
14	exp Artificial Intelligence/	118,195
15	exp Computational Biology/	213,466
16	exp Neural Networks, Computer/	35,521
17	Big Data/	1,558
18	algorithms/	270,540
19	(machine learning or big data or convolution neural network or deep learning or network analysis or bioinformatics or predictive model* or unsupervised	366,250

	learning or supervised learning or semi-supervised learning or active learning or algorithm or ai or artificial intelligence).tw.	
20	14 or 15 or 16 or 17 or 18 or 19	757,458
21	7 and 13 and 20	412
22	limit 21 to yr="2000 -Current"	410

2.5 Selection of Sources of Evidence

The selection of relevant studies will be done using forms built using the Systematic Review and Literature Review software- Distiller-SR software package (Copyright © 2008-2021, Evidence Partners Inc.) by two reviewers. Conflicts will be resolved by consensus or, if consensus cannot be reached, a third reviewer will be consulted. Authors will not be contacted for clarification or to obtain additional information on eligible studies. The first relevance screening will consist of only reviewing the title and abstract of each citation, and the second relevance screening will consist of reviewing the full text of those citations that are retained after the first screening phase. Prior to screening citations, the relevance screening forms will be pre-tested on the first 200 citations for the initial relevance screening and pre-tested on 20 citations for the data characterization forms. During this, screening questions will be refined accordingly. All relevant primary research citations and all citations screened at level 1 as 'unclear' will be forwarded to a second level full-text screening for relevance. Citations clearing level 2 screening will be advanced for data charting (for 'charting of study characteristics'). Reasons for exclusion will be captured for all citations.

First level screening of title/abstract:

1. Is the full body text (beyond title/abstract) available in English?
    - If yes, proceed to Q2.
    - If no, exclude.
    - If unclear, include citation for full-text second-level screening.
2. Does the abstract/title include machine learning, predictive modelling, or bioinformatics?
    - If yes, proceed to Q3.
    - If no, exclude.

- If unclear, proceed to Q3.

3. Does the abstract/title include influenza virus or coronaviruses?
   - If yes, proceed to Q4.
   - If no, exclude.
   - If unclear, proceed to Q4.
4. Does the abstract/title include host-range or transmission potential?
   - If yes, proceed to Q5.
   - If no, exclude.
   - If unclear, proceed to Q5.
5. Is the citation primary research?
   - If yes, include citation for full-text second-level screening.
   - If no, proceed to Q6.
   - If unclear, include citation for full-text second-level screening.
6. Is the publication a review?
   - If yes, flag as a review and exclude.
   - If no, flag as other and exclude.

Second-level full-text screening using the same first-level screening questions will be done for all citations identified as relevant or 'unclear' at the title/abstract level. Those included will be used in the final data charting.

2.6 Data charting process

Data charting will be conducted in DistillerSR ® by two reviewers working independently. The data charting form will be pre-tested by all reviewers on 20 random studies, following which modifications will be made, if necessary for question clarity. Conflicts will be resolved by consensus or, if consensus cannot be reached, a third reviewer will be consulted. Authors will not be contacted for clarification or to obtain additional information on eligible studies. If a citation describes more than one study, the data will be charted at the article level (i.e., information from all studies within an article will be extracted into a single DistillerSR® form).

2.7 Data Items

The proposed information that will be extracted from each eligible citation for data charting is summarized as follows, noting that there may be additional response options for the charting

questions as the review evolves. Description of the intended interpretation of the data items and rational for inclusion can be found in the supplementary data.

1. Publication Information:
    a. Year of publication.
    b. What is the primary author affiliation?
    c. What country/region is the primary author affiliated with?
    d. What department type is the primary author affiliated with?
    e. What is the funding source for the study?
    f. What was the publication type (i.e., conference proceeding, journal article, other)?
    g. What is the primary objective of the publication?
    h. What were limitations that the authors noted?
2. Data Gathering:
    a. Data sources
        I. Which databases/repositories were the sequences used taken from?
            - Public or Private?
            - International, national, or other?
            - Name of source
        II. How many sequences were available?
    b. Types of Sequences:
        I. Which Virus sequences were used?
            - Was the host known?
            - What were the hosts?
            - How was the host identified?
        II. How many sequences were used?
        III. What type of sequence was used?
3. Data Processing:
    a. Were sequences pre-processed prior to entry into machine learning algorithm (i.e., selection of learnability properties, no processing just direct sequences, etc.)?
    b. How was the algorithm taught (supervised, unsupervised, semi-supervised, other)?
4. Data Outcomes:

a. How were the outputs analysed?
    I. What were the outputs?
    II. Method(s) of obtaining output.
b. What were the key drivers of the host determination in the genomes?
c. How interpretable were the outputs?
d. How was the accuracy of the model determined?

The study results from eligible studies will not be extracted, as this is a scoping review.

2.8 Critical appraisal of individual sources of evidence

A critical appraisal of the literature will not be conducted, as this is a scoping review.

2.9 Synthesis of Results

Descriptive statistics will be used to summarize the findings of charted data and grouped according to their methodology and method of reporting general information and specific studies characteristics. . Summaries will be presented using a combination of tables, figures, and narrative text.

3. Funding

This work will be funded by OMAFRA and the First Canada Research Excellence Fund.

References

Abd-Alrazaq, A., Alajlani, M., Alhuwail, D., Schneider, J., Al-Kuwari, S., Shah, Z., Hamdi, M., Househ, M., 2020. Artificial intelligence in the fight against COVID-19: Scoping review. J. Med. Internet Res. 22, 1–18. https://doi.org/10.2196/20756

Arksey, H, & O'Malley, L. 2005. Scoping studies: towards a methodological framework, International Journal of Social Research Methodology, 8:1, 19-32. doi:10.1080/1364557032000119616

Ashford, R.W., 2003. When Is a Reservoir Not a Reservoir? [4]. Emerg. Infect. Dis. 9, 1495–1496. https://doi.org/10.3201/eid0911.030088

Dolan, P.T., Whitfield, Z.J., Andino, R., 2018. Mapping the Evolutionary Potential of RNA Viruses. Cell Host Microbe 23, 435–446. https://doi.org/10.1016/j.chom.2018.03.012

Geoghegan, J.L., Holmes, E.C., 2017. Predicting virus emergence amid evolutionary noise. Open Biol. 7. https://doi.org/10.1098/rsob.170189

Grange, Z, Goldstein, T, Johnson, C, Anthony, S, Gilardi, K, Daszak, P, Olival, K, O’Rouke, T,

Murray, S er al. 2021. Ranking the risk of animal-to-human spillover for newly discovered viruses, Proceedings of the National Academy of Sciences of the United States of America 118(15) e2002324118. DOI: 10.1073/pnas.2002324118

Haydon, D.T., Cleaveland, S., Taylor, L.H., Laurenson, M.K., 2002. Identifying reservoirs of infection: A conceptual and practical challenge. Emerg. Infect. Dis. 8, 1468–1473. https://doi.org/10.3201/eid0812.010317

Lee, B, Smith, DK, Guan, Y. 2021. Alignment free sequence comparison methods and reservoir host prediction, Bioinformatics. 8:btab338. doi: 10.1093/bioinformatics/btab338. Epub ahead of print. PMID: 33964132; PMCID: PMC8135978.

Tricco, AC, Lillie, E, Zarin, W, O'Brien, KK, Colquhoun, H, Levac, D, Moher, D, Peters, MD, Horsley, T, Weeks, L, Hempel, S, et al. 2018. PRISMA extension for scoping reviews (PRISMA-ScR): checklist and explanation, Annals of Internal Medicine 169(7): 467-473. doi:10.7326/M18-0850

Viana, M., Mancy, R., Biek, R., Cleaveland, S., Cross, P.C., Lloyd-Smith, J.O., Haydon, D.T., 2014. Assembling evidence for identifying reservoirs of infection. Trends Ecol. Evol. 29, 270–279. https://doi.org/10.1016/j.tree.2014.03.002

Table 1.Supplemental Materials -Definitions for the Scoping Review:

Section 2.7 Data Items Definitions and Rationale:

1.	a.	The year of publication is the year in which the research was published. This information can outline the development of this area of research.
	b.	The affiliation of the primary author (first author) indicates the industry that the author is working for such as university, government, private corporation, etc. This can help determine what type of researchers are interested in this area of research.
	c.	The country of affiliation indicates the country the author is publishing their work from and likely conducting their research. This can help determine what countries are interested in this area of research.
	d.	The department of the primary author describes what area of research the author works in. This will help formulate who is investigating this area of research.
	e.	The funding source indicates the industry that the funding for the research is derived from such as university, government, private corporation, etc. This can help determine what type of industries are interested in this area of research.

	f.	The publication type refers to the type of publication (ex. Journal article or conference proceeding). This can help identify what stages the body of evidence is at.
	g.	The primary objective of the paper will be pulled from the abstract or introduction of the paper and will state what the author's primary area of interest was. This will help show if the proposed scoping review question is the main area of research of interest or if it is a by-product. It will also help identify which specific viruses are of interest in this research alongside question 2. b.I .
	h.	The limitations of the paper are any limitations the researchers may have faced when completing their research. For example, lack of genome data available, vagueness regarding common definitions, etc. This question provides insights into how this research and field can be developed.
2.	a. I	There are numerous sources for genome sequences available which will be indicated by collecting the source name. This availability can be classified into those that are publicly available versus those that are not. This information will indicate where most researchers are obtaining the information, they are using for their host determination algorithms and give better insight on where sequences may lack in some models.
	a. II	The number of sequences available is the amount of information the researcher had available to them dependent on which databases they used, which virus, host, etc. The number of sequences available will also help in the reflection of the accuracy of the machine learning models, those with a greater number of sequences available for training may be more reliable.
	b. I	The virus and host sequences that were used are the species of host(s) used in the researcher's model and the virus(es). Also, how was the host determined; was it already known, or was it predicted using the researchers' methods or by some other method. This information is key in answering the PICO elements of the stated question.
	b. II	How many sequences were used refers to the number of sequences used for each part of the model- virus(es), host(s). This number may be equivalent to the number of sequences available. The number of sequences used can reflect the extent of the model design.

	b. III	The type of sequence used refers to the type. For example, if the sequence has been defined at the nucleotide level. This information will help formulate a clearer understanding of the research methodology.
3.	a.	When developing models often sequences will be processed to be able to make the sequences more learnable for the algorithm or more comparable to other sequences. However, in doing so some data may be lost or the reliability of the model may be hindered. Identifying the techniques used for pre-processing will help develop a better understanding of how the models are developed and some of their limitations.
	b.	How the model was taught refers to the method of teaching the machine learning algorithm. Three common methods are:<br>(I) Supervised Learning: This approach can be taken when the input and output values of data are both known. Using this information, a model is developed and then used to predict future outcomes.<br>(II) Unsupervised Learning: This is used with a given input source and an unknown output. The algorithm will use tools to develop an output it sees as appropriate and categorically correct.<br>(III) Semi-Supervised Learning: This approach combines both supervised and unsupervised learning techniques. By using a smaller supervised input with known output to guide the larger input source without a known output to develop similar categorizations.<br>Knowing how researchers have trained their algorithms will give further insight into the future development of machine learning tools for virus-host information.
4.	a. I	What type of outputs were given for the sequence analysis? Identify how the authors have decided to display their results.
	a. II	Methods that were used to create the output and analyse the output of the given machine learning data.
	b.	The key drivers refer to the main parts of the sequence or main parameters in the given model that had the greatest outcome on the given output of the model. This will help show the most significant effects on host identification.
	c.	The interpretability of the model reflects the model's complexity. The model complexity is in reference to the number of parameters that have been used to define the model. The interpretability of the data also reflects the ability to identify

		which factors have the greatest effect on the identification of virus hosts. The complexity of the model often directly reflects the method by which the model was taught as identified in question 3. b. For example, a supervised learning model will usually have more interpretable results than unsupervised methods.
	d.	How was the accuracy of the model determined? The accuracy of the model's ability to identify the hosts of a virus is determined by the author how have they defined the accuracy. This information provides how many different measurements of accuracy exist and how the accuracy of a model can be defined.
